# Heterogeneity in conservation of multifunctional partner enzymes with meiotic importance, CDK2 kinase and BRCA1 ubiquitin ligase

**DOI:** 10.7717/peerj.12231

**Published:** 2021-09-27

**Authors:** Sergey Matveevsky, Tatiana Grishaeva

**Affiliations:** Laboratory of Cytogenetics, Vavilov Institute of General Genetics, Russian Academy of Sciences, Moscow, Russia

**Keywords:** Meiosis, Enzymes, CDK2, BRCA1, Synaptonemal complex, Bioinformatics, Immunocytochemistry

## Abstract

The evolution of proteins can be accompanied by changes not only to their amino acid sequences, but also their structural and spatial molecular organization. Comparison of the protein conservation within different taxonomic groups (multifunctional, or highly specific) allows to clarify their specificity and the direction of evolution. Two multifunctional enzymes, cyclin-dependent kinase 2 (CDK2) and BRCA1 ubiquitin ligase, that are partners in some mitotic and meiotic processes were investigated in the present work. Two research methods, bioinformatics and immunocytochemical, were combined to examine the conservation levels of the two enzymes. It has been established that CDK2 is a highly conserved protein in different taxonomic lineages of the eukaryotic tree. Immunocytochemically, a conserved CDK2 pattern was revealed in the meiotic autosomes of five rodent species and partially in domestic turkey and clawed frog. Nevertheless, variable CDK2 distribution was detected at the unsynapsed segments of the rodent X chromosomes. BRCA1 was shown to be highly conserved only within certain mammalian taxa. It was also noted that in those rodent nuclei, where BRCA1 specifically binds to antigens, asynaptic regions of sex chromosomes were positive. BRCA1 staining was not always accompanied by specific binding, and a high nonspecificity in the nucleoplasm was observed. Thus, the studies revealed different conservation of the two enzymes at the level of protein structure as well as at the level of chromosome behavior. This suggests variable rates of evolution due to both size and configuration of the protein molecules and their multifunctionality.

## Introduction

Protein evolution is central to research in areas such as comparative genomics and proteomics, molecular evolution, and structural biology. The evolution of proteins is often accompanied by the modification of the primary amino acid sequence, which is directly related to changes in the nucleotide sequence. One of the methods for studying the evolution of various molecules is to compare the homologous relationships of nucleotide and/or amino acid sequences in different taxonomic groups. Combining the methods of computational proteomics and immunocytochemical studies of proteins *in situ* makes it possible to assess the evolution of proteins as universal or specific.

Eukaryotic cells may undergo two major types of cell division, namely mitosis and meiosis. Meiosis is a special two-step process of cell-nuclear division that reduces the chromosome number by half, resulting in the production of haploid gametes. This process requires segregation of homologous chromosomes in Meiosis I (reduction division) and sister chromatids in Meiosis II (equational division). Prophase I, as the most complex and extended stage of Meiosis I, includes unique processes: pairing between homologs, formation of programmed DNA double-strand breaks (DSBs), homologous recombination leading to crossover formation, synaptonemal complex (SC) assembly and disassembly, and chromatin reorganization (Roeder, 1997; Page & Hawley, 2003; Page & Hawley, 2004; Turner et al., 2005). The SC is a tripartite nucleoprotein structure consisting of two parallel lateral elements (LEs) that are linked together in a “zipper”-like mode *via* the central element (CE) and numerous perpendicularly running transverse filaments (TFs) (Moses, 1968; Zickler & Kleckner, 1998).

The hundreds of proteins operating during meiosis can be classified as either structural (*e.g.*, cohesins, condensins, histones, and SC proteins) or regulatory (*e.g.*, separase and ATR kinase) in terms of their enzymatic activity. The conservation of proteins involved in meiotic processes is highly variable (Grishaeva, 2018; Grishaeva & Bogdanov, 2018). For instance, RAD51, DMC1, and MLH1 enzymes are more evolutionary conserved than structural meiotic proteins (RAD21, REC8, and HORMA-domain proteins) (Grishaeva & Bogdanov, 2018). The many enzymes involved are either meiosis-specific proteins or universal proteins involved in many cellular processes; the latter group includes cyclin-dependent kinase 2 (CDK2) and the E3-ubiquitin-protein ligase BRCA1 (breast cancer type 1 susceptibility protein) whose activity is coupled with a myriad of other protein classes as well as each other. Thus, comparison of the conserved nature of these enzymes may be of particular interest.

CDK2, also known as cell division protein kinase 2, belongs to the family of serine/threonine protein kinases that are involved in the regulation of the eukaryotic cell cycle (Morgan, 1997; Morgan, 2006). More than 20 CDKs are currently known in mammals (Malumbres et al., 2009), however, only some of these are directly involved in cell cycle initiation and processing (Malumbres, 2014). CDKs are also involved in the repair of DNA DSBs (Hydbring, Malumbres & Sicinski, 2016). CDK2 acts together with various cyclins to form specific heterodimeric complexes (Morgan, 1997). These complexes then phosphorylate proteins that control cell entry into the G1 phase, DNA synthesis in the S phase, and chromosome segregation during mitotic anaphase (Morgan, 1997; Hydbring, Malumbres & Sicinski, 2016). The CDK2-cyclin complex also phosphorylates BRCA1 (Esashi et al., 2005). CDK2 is required for precise synapsis of homologs, meiotic recombination, and sex body formation (Viera et al., 2009). CDK2 also maintains histone H3 in a methylated state (H3K27me3), providing gene silencing (information from the GeneCards database). It has also been reported that the precise regulation of CDK2 kinase activity in male germ cell development is critical for the transition of gonocytes to spermatogonia (Singh & Schimenti, 2015; Singh et al., 2019).

BRCA1 plays an important role in the repair of DNA DSBs and DNA recombination, both in somatic and germ cells (Marcon & Moens, 2005). BRCA1 is essential for the transition from the G2 stage of the cell cycle to mitosis (Yarden et al., 2002), which requires phosphorylation of BRCA1 by the Aurora-A kinase (Ouchi et al., 2004). BRCA1 is a tumor suppressor (Stefansson & Esteller, 2012), and patients with *brca1* gene mutations are at risk of developing various cancers (Claus, Risch & Thompson, 1991; Mersch et al., 2015). During meiosis, BRCA1 first appears in the early zygotene, localizing on still unpaired chromosomal axes (Scully et al., 1997; Marcon & Moens, 2005). At the pachytene stage, BRCA1 recruits PI3K-like kinase ATR to unpaired DNA for phosphorylation of H2AX histone, leading to silencing (suppression) of the corresponding chromatin sites (MSUC and MSCI) (Turner et al., 2004; Turner et al., 2005; Garcia-Cruz et al., 2009). Additionally, BRCA1 interacts with BRCA2 and colocalizes with RAD51 at unpaired chromosome sites, controlling meiotic recombination (Scully et al., 1997; Sy, Huen & Chen, 2009).

It has been found previously that, in some models, the BRCA1 protein exhibits low conservation levels even within the vertebrates (Grishaeva, 2018). BRCA1 orthologs were absent in the databases for fungi and invertebrates (GeneCards). A similarity of orthologs was found only in humans and mice. Most of the immunocytological studies of BRCA1 have been performed on human and mouse meiotic chromosomes. However, in a recent work by Li et al. (2018), the colocalization of BRCA1 (BRC-1) with RAD-51 within the SC of the *Caenorhabditis elegans* was shown.

The objectives of this study were to perform a comparative analysis on the architecture of amino acid sequence motifs and conservation levels of CDK2 and BRCA1 partner enzymes in various eukaryotic species, using bioinformatics methods and localization of these proteins *via* immunostaining and fluorescent microscopy of spermatocytes of available vertebrate species (Fig. S1). This study expands on our previous papers (Grishaeva, 2018; Matveevsky et al., 2021). This is the first study to provide results on the conserved primary structure of CDK2 in different phylogenetic lineages of eukaryotes, as well as the first to analyze the distributions of CDK2 and BRCA1 in pachytene spermatocytes of some rodents, domestic turkey, and clawed frog, and graphically represent the distributions of these proteins along the X chromosomes (CDK2 and BRCA1 immuno-profiles). Turkey SCs are presented for the first time.

## Materials & Methods

### Characterization of ortholog dataset

Orthologs of enzymes were studied using bioinformatic approaches. Totally 20 amino acid sequences of CDK2 orthologs (verified or predicted) across 16 vertebrate species were analyzed: human *Homo sapiens* (further referred to as Hs, protein ID – CDK2_HUMAN), house mouse *Mus musculus* (Mm_b, CDK2_MOUSE, isoform alpha), the laboratory rat *Rattus norvegicus* (Rn, NP_955795.1), Chinese hamster *Cricetulus griseus* (Cg, CDK2_CRIGR), golden hamster *Mesocricetus auratus* (Ma, XP_012975721.1, isoform X1), prairie vole *Microtus ochrogaster* (Mo, XP_005371228.1, isoform X2), Upper Galilee mountains blind mole rat *Nannospalax galili* (Ng, XP_008846910.1, isoform X2), common shrew *Sorex araneus* (Sa, XP_004601649.1, isoform X2), chicken *Gallus gallus* (Gg, NP_001186786), zebra finch *Taeniopygia guttata* (Tg, XP_032600706.1), Ring-necked pheasant *Phasianus colchicus* (Pco, XP_031465194.1), green anole *Anolis carolinensis* (Ac, predicted protein XP_008112629), African clawed frog *Xenopus laevis* (Xl, NP_001084120.1), American bullfrog *Lithobates catesbeianus* (Lc, C1C4M4_LITCT), two-lined caecilian *Rhinatrema bivittatum* (Rb, XP_029450626.1), zebrafish *Danio rerio* (Dr, NP_998571); proteins of two invertebrates species, fruit fly *Drosophila melanogaster* (Dm, NP_732544) and roundworm (nematode) *Caenorhabditis elegans* (Ce, WormBase ID: CE51951); proteins of thale cress *Arabidopsis thaliana* (At, NP_566911, cell division control 2, synonym CDK2); and baker’s yeast *Saccharomyces cerevisiae* (Sc, NP_009718). Protein of fission yeast *Schizosaccharomyces pombe* (Sp, NP_595629) was analyzed partially (this predicted ortholog is mentioned in GeneCards database but is removed from NCBI database). Proteins sizes vary from 294 to 368 amino acids (aa). We were not able to find any orthologs of CDK2 of turkey *Meleagris gallopavo* in databases but the behavior of this protein was studied here using the immunocytochemical method.

Twenty-five orthologs of BRCA1 ubiquitin ligase (verified or predicted) were analyzed. We used 20 sequences found in vertebrates species: human *Homo sapiens* (Hs, BRCA1_HUMAN, isoform 1), house mouse *Mus musculus* (Mm, BRCA1_MOUSE), the laboratory rat *Rattus norvegicus* (Rn, BRCA1_RAT), prairie vole *Microtus ochrogaster* (Mo, A0A7G0XG68_MICOH, fragment), common shrew *Sorex araneus* (Sa, Q8WMT5_SORAR, fragment), Upper Galilee mountains blind mole rat *Nannospalax galili* (Ng, XP_029413275.1), Chinese hamster *Cricetulus griseus* (Cg, G3HM05_CRIGR), golden hamster *Mesocricetus auratus* (Ma, A0A3Q0CPK7_MESAU), the striped field mouse *Apodemus agrarius* (B0FT01_APOAG, fragment), and three species of mole vole: *Ellobius lutescens* (El, QOE89012.1, partial), *Ellobius talpinus* (Etal, QOE89036.1, partial), *Ellobius tancrei* (Etan, QOE89029.1, partial); proteins of chicken *Gallus gallus* (Gg, NP_989500.1), zebra finch *Taeniopygia guttata* (Tg, H0YXC9_TAEGU), Ring-necked pheasant *Phasianus colchicus* (Pco, A0A669PZE1_PHACC); proteins of three-toed box turtle *Terrapene carolina triunguis* (Tct, A0A674JGI1_TERCA); proteins of the African clawed frog *Xenopus laevis* (Xl, AAL13037.1) and the Western clawed frog *Xenopus tropicalis* (Xt, XP_012807962.2), and two-lined caecilian *Rhinatrema bivittatum* (Rb, XP_029429046.1); and coelacanth *Latimeria chalumnae* (Lch, H3AAR9_LATCH). In addition, BRCA1 orthologs of three invertebrate species were analyzed, proteins of owl limpet *Lottia gigantea* (Lg, V4AQT9_LOTGI), roundworm (nematodes) *Caenorhabditis elegans* (Ce, BRCA1_CAEEL), purple sea urchin *Strongylocentrotus purpuratus* (Spu, A1YZ82_STRPU) and two plant proteins, those of maize *Zea mays* (Zm, A0A1D6E647_MAIZE) and rice *Oryza sativa* (Os, Q7XPZ2_ORYSJ) (Fig. S1). Databases do not contain any orthologs of BRCA1 from fungi. Full-size proteins vary in length from 612 to 2,942 aa, partial ones—from 376 to 923 aa.

### Bioinformatic analysis of proteins

Comparative studies of proteins were made by methods of computational biology. The parameters of protein primary sequences were analyzed (the presence of common functional domains, the set and location of conserved motifs, isoelectric points), as well as secondary structure (the probability of coiled-coils formation). The unrooted phylogenetic trees were constructed using the Constraint-based Multiple Protein Alignment Tool (COBALT) from the NCBI tool package (http://www.ncbi.nlm.nih.gov/tools/cobalt/cobalt.cgi?CMD=Web). Parameters: maximal sequence difference 0.9, other parameters—by default. At the final stage of constructing trees, the Neighbor Joining algorithm was employed. Proteins are listed in figure captions. Amino acid sequences of proteins were found in four databases, GeneCards (https://www.genecards.org/), UniProKB (http://www.uniprot.org/), NCBI (http://www.ncbi.nlm.nih.gov/guide/) or WormBase (http://www.wormbase.org/). The presence of conserved functional domains was detected by the CDART program (http://www.ncbi.nlm.nih.gov/Structure/cdd/wrpsb.cgi?), and the set and location of amino acid conserved motifs by the MEME program (https://meme.nbcr.net/). Original figures of amino acid motifs are available in the Mendeley Data (see the link in the Data availability section). To determine the secondary structure of orthologs, the COILS program was used (https://embnet.vital-it.ch/software/COILS_form.html), the isoelectric points of proteins (pI) were detected using the Compute pI/Mw tool (http://web.expasy.org/compute_pi/).

**Figure 1:**
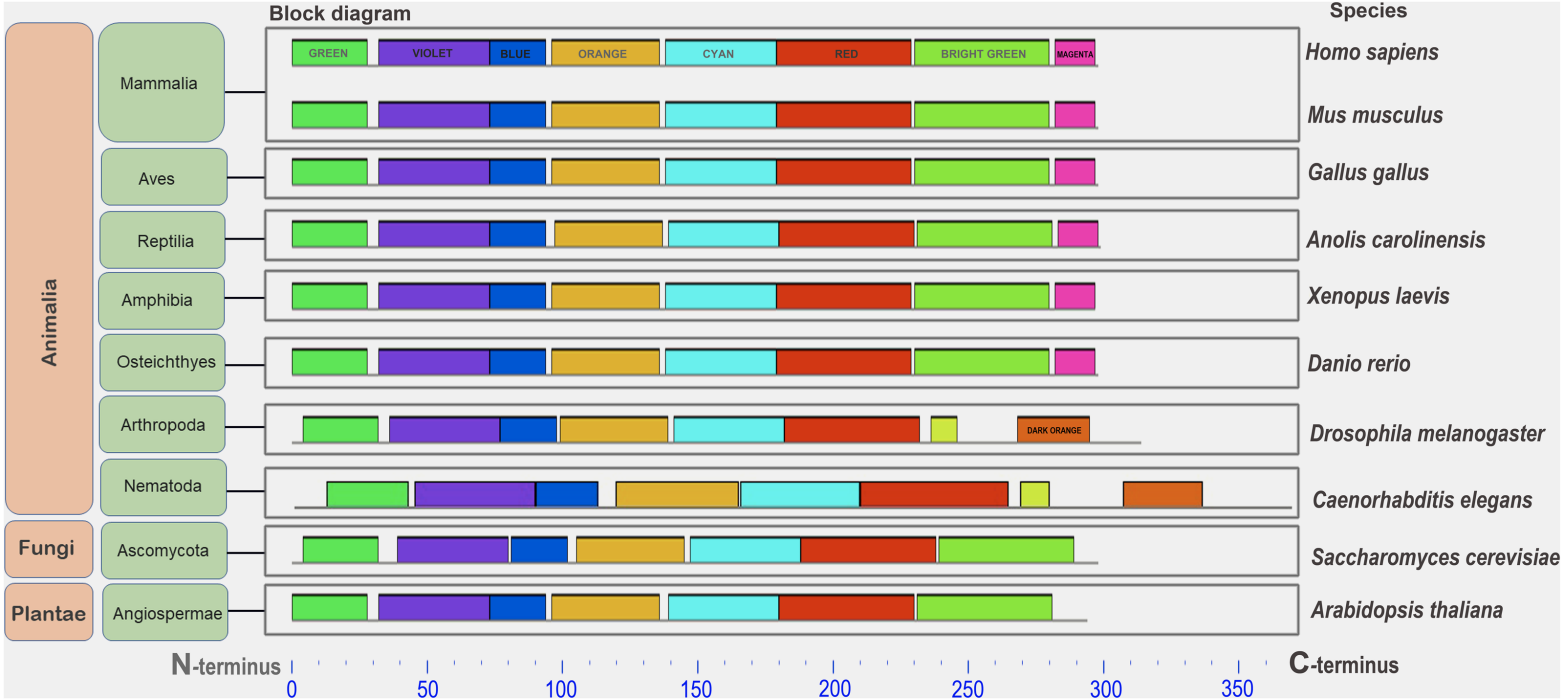
A set of conserved amino acid motifs in CDK2 proteins from proteomes representing a wide range of eukaryotes. Human (*H. sapiens*), mouse (*M. musculus*) (mammals); chicken (*G. gallus*) (birds); green anole (*A. carolinensis*) (reptile); clawed frog (*X. laevis*) (amphibia); zebrafish (*D. rerio*) (bony fish); fruit fly (*D. melanogaster*) (insects); roundworm (*C. elegans*) (nematodes); yeast (*S. cerevisiae*) (fungi); and Arabidopsis (*A. taliana*) (plants). Identical motifs are indicated by rectangles of the same color and size. The N and C termini of proteins are indicated. Distant taxa have similar motif architecture.

A conserved functional domain is a part of a protein that sustains its function. This fragment of protein molecule is shared by the majority of proteins included in the given protein family and has a similar but nonidentical amino acid sequence in family members. A conserved amino acid motif is a small fragment of protein molecule in or outside the functional domain which is shared by some or all members of given protein family (Fig. S2). It also has highly similar amino acid sequence. Protein similarity can vary significantly when comparing whole molecules. However, the similarity in individual parts of a protein molecule can serve as an important indicator of conservation of protein fragments required to perform a specific function. Conserved motifs are presented in the figures as color rectangles along the amino acid sequences of proteins. The rectangles of the same size and color represent the same motifs in all proteins presented in the given figures (Fig. 1, Figs. S3–S5).

### Animals for immunocytochemical analyses

Among the objects of immunocytochemical studies were males of different vertebrate species: the laboratory rat *Rattus norvegicus* (from the vivarium of the VIGG RAS), the common vole *Microtus arvalis* (internal number: #MA001), the bank vole *Clethrionomys glareolus* (#CG01), the pygmy wood mouse *Sylvaemus uralensis* (#SU01) (voles and wood mouse were presented by V.M. Malygin), the Asian wood mouse *Apodemus peninsulae* (AP01; presented by Yu.M. Borisov), subterranean rodent—the northern mole vole *Ellobius talpinus* (#27041, #2737; presented by I.Yu. Bakloushinskaya), bird—the domestic turkey *Meleagris gallopavo* (MG001; presented by M.M. Atsaeva) and amphibia—the African clawed frog *Xenopus laevis* (#001, #002; presented by S. Stolyarov).

Animals were treated according established international rules (Stokes, 2000) and international protocols, such as the Guidelines for Humane Endpoints for Animals Used in Biomedical Research. All experimental protocols, including euthanasia of animals by cervical displacement under isoflurane-induced anesthesia, were approved by the Ethics Committees for Animal Research of the Vavilov Institute of General Genetics (order No. 3 of November 10, 2016) in strict accordance with Regulations for Laboratory Practice in Russian Federation. All efforts were aimed at reducing the number and suffering of experimental animals while meeting the needs of the experiment.

### Meiotic chromosome spreading and immunostaining procedure

The meiotic chromosome/synaptonemal complex spreads were prepared and fixed by the method of Peters et al. (1997) in the modification by Page et al. (2003).

Immunostaining of AEs and LEs of the SCs was performed using rabbit antibodies against the C-terminus of human SYCP3 protein (ab15093, dilution 1: 500, Abcam, Cambridge, UK). CDK2 localization was detected using mouse antibodies against the full-length human CDK2 protein (sc-6248, 1:50–1:250, Santa Cruz Biotechnology Inc., Santa Cruz, CA, USA). The BRCA1 localization was identified applying mouse antibodies against the epitope within 304 amino acids of the N-terminal region of the human BRCA1 protein (ab16781, 1:20, Abcam, Cambridge, UK). The primary antibodies were diluted in an antibody dilution buffer (ADB: 3% bovine serum albumin—BSA, 0.05% Triton X-100 in phosphate-buffered saline (PBS)). Goat anti-rabbit IgG conjugated with Alexa Fluor 488 (#A11008, Invitrogen, Carlsbad, CA, USA), goat anti-mouse IgG Alexa Fluor 555-conjugated (ab150118, Abcam, Cambridge, UK) (diluted 1:200–800) was used as secondary antibodies. Slides were washed in PBS and putting into Vectashield medium with 4′,6-diamidino-2-phenylindole (DAPI) (Vector Laboratories, Burlingame, CA, USA). The preparations were examined under an AxioImager D1 fluorescence microscope (Carl Zeiss, Jena, Germany) equipped with an Axiocam HRm CCD camera (Carl Zeiss), and image-processing AxioVision Release 4.6.3. software (Carl Zeiss, Germany) (using 100× magnification). Immunostaining was performed using the method of single-round immunostaining with two antibodies according the established protocol (Kolomiets, Matveevsky & Bakloushinskaya, 2010; Matveevsky, Bakloushinskaya & Kolomiets, 2016; Matveevsky et al., 2021). The first step included SYCP3/CDK2 or SYCP3/BRCA1 staining, and then the second step by incubation with the corresponding secondary antibodies. Uncropped micro photos are available in the Mendeley Data (see the link in the Data availability section).

The negative control consisted of (1) overnight incubation with an antibody dilution buffer (ADB, 10 μl) without any antibodies and (2) overnight incubation with a secondary goat anti-mouse IgG Alexa Fluor 555-conjugated antibody (ab150118, 10 μl, 1:200) in a humid chamber. After incubation, the slides were washed in PBS 3 times for 2 min and then embedded in Vectashield medium with DAPI. The control was carried out on three species (*Clethrionomys glareolus*, *Sylvaemus uralensis*, *Meleagris gallopavo*). Then the slides were examined under a microscope. The exposure was the same as for the CDK2 and BRCA1 signals. The microscope used fluorescent filters from Carl Zeiss: Filter set 01 (blue), Filter set 38HE (green), and Filter set 43HE (red). The results are shown in Figs. S6 and S7. It is worth noting that the expression/localization of the assessed proteins may not entirely reflect an accurate scenario, since the anti-BRCA1 and CDK2 antibodies employed in the study, both raised against the human proteins, could not be tested for specificity in the null mutants of the relative organisms.

### CDK2 and BRCA1 immuno-profiles (qualitative analysis)

The use of the term “profile” or “immuno-profile” means the qualitative features of the distribution of immunosignals and is in no way associated with any characteristics of gene expression or any protein activities. For constructing immuno-profiles, the length of the asynaptic region of the X and/or Y chromosomes was divided into 10 equal segments. In each of these parts, the CDK2 or BRCA1 signals were calculated for all sex bivalents. If there was at least one signal per segment, then this part was considered positive (see Raw data in Data availability section). For this qualitative analysis, sex bivalents with clearly identified X and Y chromosomes and without associations were considered (during the transition from early to mid pachytene) to adequately assess the position of signals in each sex chromosome. The profiles were built using the Chart option in Microsoft Excel 2007.

## Results

### The domain structure and other parameters of CDK2 proteins

The sizes of all studied proteins were very similar: in humans, mice, fish, chicken, and yeast Sc, the CDK2 proteins were 298 aa; in yeast Sp and frog they were 297 aa; in the lizard it was 299 aa; and in Arabidopsis, it was 294 aa (*i.e.,* in vertebrates, plants, and yeast the protein ranged from 294 to 299 aa). In invertebrates, the proteins were slightly longer (314 aa for Drosophila and 338 aa in a nematode). Almost the entire protein molecule is occupied by one functional domain, specifically STKc_CDK2_3 (serine/threonine kinase, cyclin-dependent kinase 2 and 3 from the PKc_like superfamily) (Fig. S2). In addition to this domain, numerous active sites have been identified, in particular, sites for binding to ATP and other polypeptides. In Arabidopsis, another functional domain, PLN00009 (cyclin-dependent kinase A, provisional), was identified. This domain is annotated only for green plants (Viridiplantae). No active sites were found in this protein. It was assumed that the Arabidopsis protein was very different from other orthologs.

A secondary structure of the protein, *i.e.,* the ability of the protein to form an alpha-helical configuration, was not detected in any studied species, including the yeast Sp. Protein isoelectric points (pIs) ranged from 6.40 in Arabidopsis to 9.08 in the chicken Gg. Among vertebrates, the pIs were close, ranging from 8.64 to 9.08. Meanwhile, according to this parameter, the orthologs were not as conserved.

### Conserved amino acid motifs in CDK2 proteins

In all analyzed distinct species, except for the yeast Sp (not analyzed here), a similar set of conserved amino acid motifs was found (Fig. 1). The architecture of motifs was identical among vertebrates. Nematode (Ce) and Drosophila (Dm) had different motifs at the C terminus of the protein (yellow and orange rectangles instead of light green and purple). Yeast (Sc) and Arabidopsis (At) lacked one small motif (purple rectangle) in the C-terminus of the CDK2. Nevertheless, all CDK2 orthologs were highly conserved.

In the second step of the analysis, high kinase conservation was confirmed for three vertebrate groups, specifically mammals, birds, and amphibians (see Fig. S3). In the third step of the analysis, an identical motif set was identified across all mammals studied (humans, six rodent species, and insectivore, see Fig. S4).

### Immunocytological analysis of CDK2 localization in pachytene spermatocytes of different species

**Figure 2:**
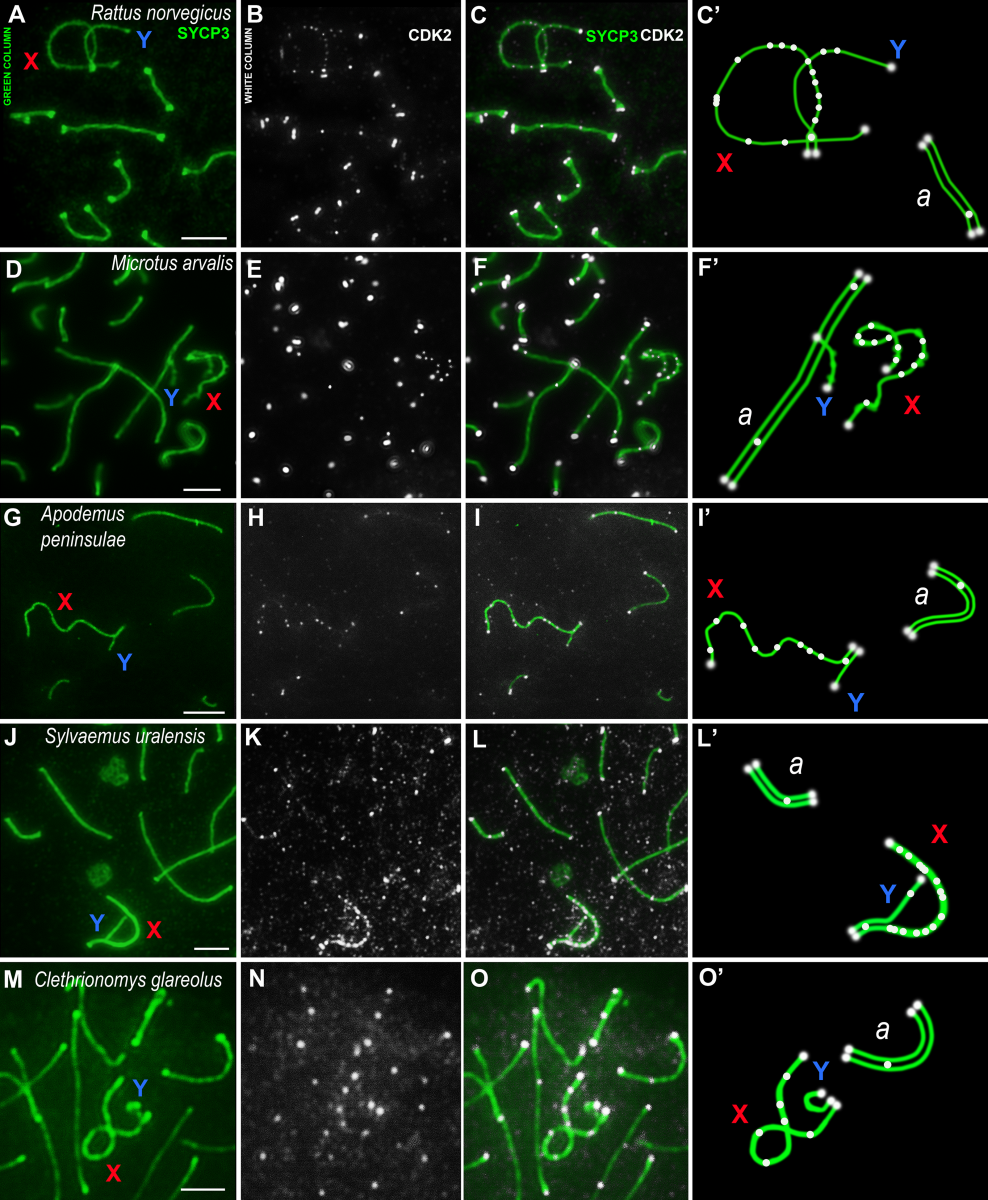
Rodent pachytene spermatocytes. SCs were immunostained using antibodies to SYCP3 protein (green), and CDK2 using antibodies to CDK2 (white). Parts of spermatocytes with autosomal SCs (designated as *a*) and sex bivalents of the rodents are present: (A–C) rat, *Rattus norvegicus*; (D–F) vole, *Microtus arvalis*; (G–I) wood mouse, *Apodemus peninsulae*; (J–L) wood mouse, *Sylvaemus uralensis*; (M–O) vole, *Clethrionomys glareolus*. Schemes of the sex bivalents and one of the autosomal SCs are shown in C’, F’, I’, L’, and O’. The female sex chromosome is marked with a red “X”. The male sex chromosome is marked with a blue “Y”. Scale bar = 5 µm.

**Figure 3:**
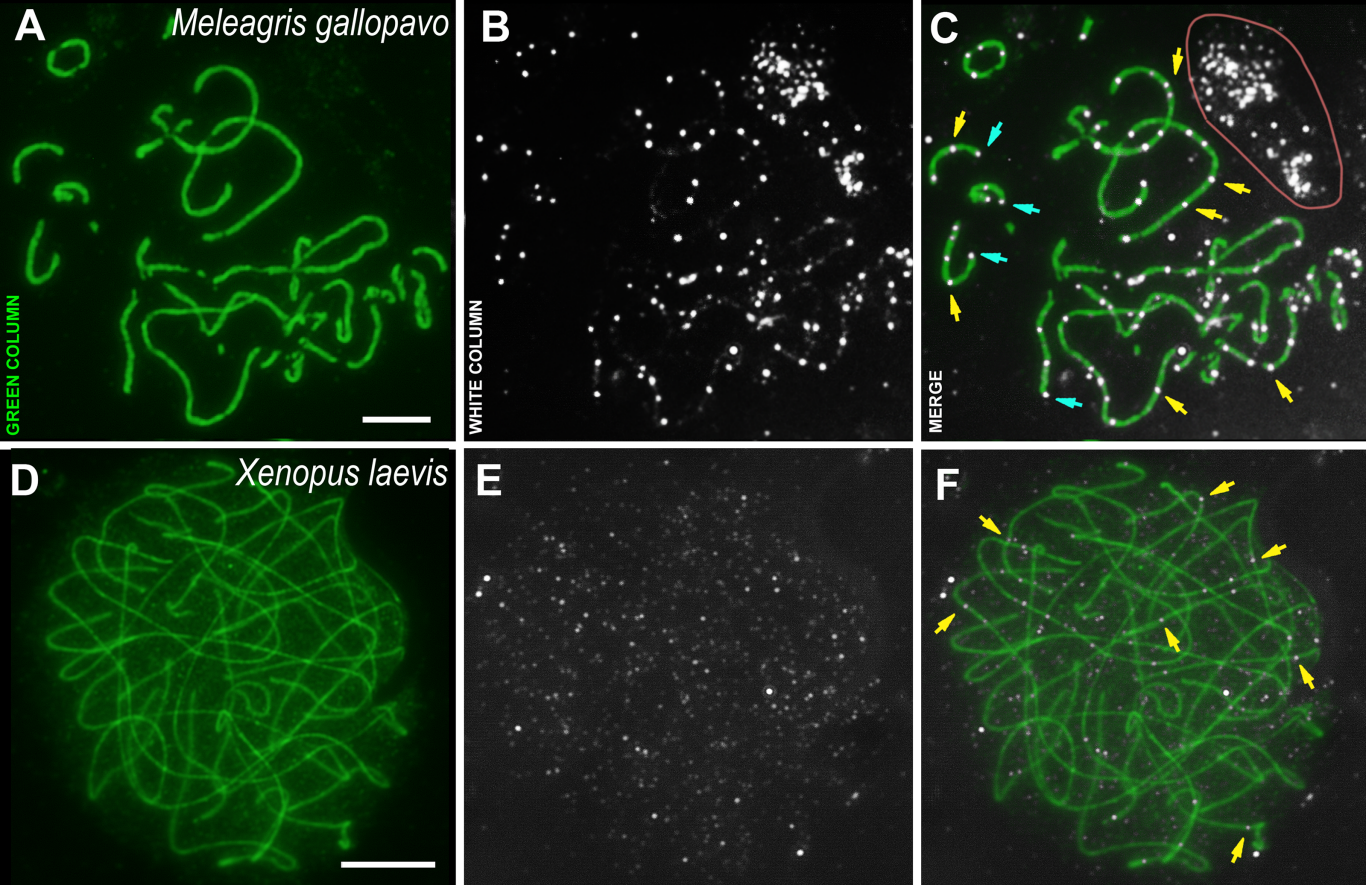
Bird and amphibia pachytene spermatocytes. SCs were immunostained using antibodies to SYCP3 protein (green), and CDK2 using antibodies to CDK2 (white). Yellow arrows indicate some interstitial CDK2 signals. Powder-blue arrows indicate telomeric CDK2 signals. (A–C) Turkey, *Meleagris gallopavo*. The light pink line outlines the CDK2-dots cloud outside the cell (outside the DAPI staining, see Data set), which was observed near all turkey nuclei of the spermatocytes; (D–F) frog, *Xenopus laevis*. Scale bar = 5 µm.

Using mouse antibodies to CDK2, the kinase distributions in the meiotic chromosomes of five rodent species, three of which were presented here for the first time (Fig. 2), as well as the domestic turkey, *M. gallopavo*, and the frog, *X. laevis*, were studied in more details (Fig. 3). CDK2 signals have different localization patterns in autosomal and sex bivalents. In the autosomal SCs of five rodent species, CDK2 signals were located by large dots in telomeric regions and by 1–2 smaller dots in interstitial sites (Figs. 2A–2O). Heteromorphic sex bivalents of the rat, *R. norvegicus*, the vole, *C. glareolus*, and the wood mice, *A. peninsulae* and *S. uralensis,* are represented by long X and short Y chromosomes, which are synapsed in a short homology region called the pseudoautosomal region (PAR) (Burgoyne, 1982). In *M. arvalis* only, the heterochromosomes do not have a PAR, and thus are usually referred to as asynaptic (Ashley, Jaarola & Fredga, 1989). For rodent’s XY, CDK2 signals were localized in the telomeric regions; as a rule, there was a single signal in the synaptic region, from 0 to 2–3 foci in the asynaptic Y axis, and a large number of CDK2 dots along the asynaptic *X* axis (Figs. 2C, 2C’, 2F, 2F’, 2I, 2I’, 2L, 2L’, 2O, 2O’).

The male turkey and frog sex chromosomes are homomorphic (Schmid et al., 2005; Schmid & Steinlein, 1991; Uno et al., 2008) and behave as autosomes in prophase I (Loidl & Schweizer, 1992; Matveevsky, Stolyarov & Kolomiets, 2019). In the turkey meiotic chromosomes, CDK2 signals were found specifically bound to interstitial sites, whereas foci were less frequent in the telomeric regions when compared to rodents (Figs. 3A–3C). Weak CDK2 signals were also visualized along some turkey SCs (Figs. 3B, 3C). CDK2 staining was carried out on two individuals of *X. laevis*. In the first animal, no specific signals were detected (antibody dilution - 1: 250). Therefore, we used CDK2 antibodies with a weaker dilution (1:50) for the second frog specimen and, in this case, weak (miniature) interstitial CDK2 signals were detected within the SCs, as well as a lot of small nonspecific CDK2 dots in the nucleoplasm (Figs. 3D–3F). There were no signals detected in the telomeric regions.

**Figure 4:**
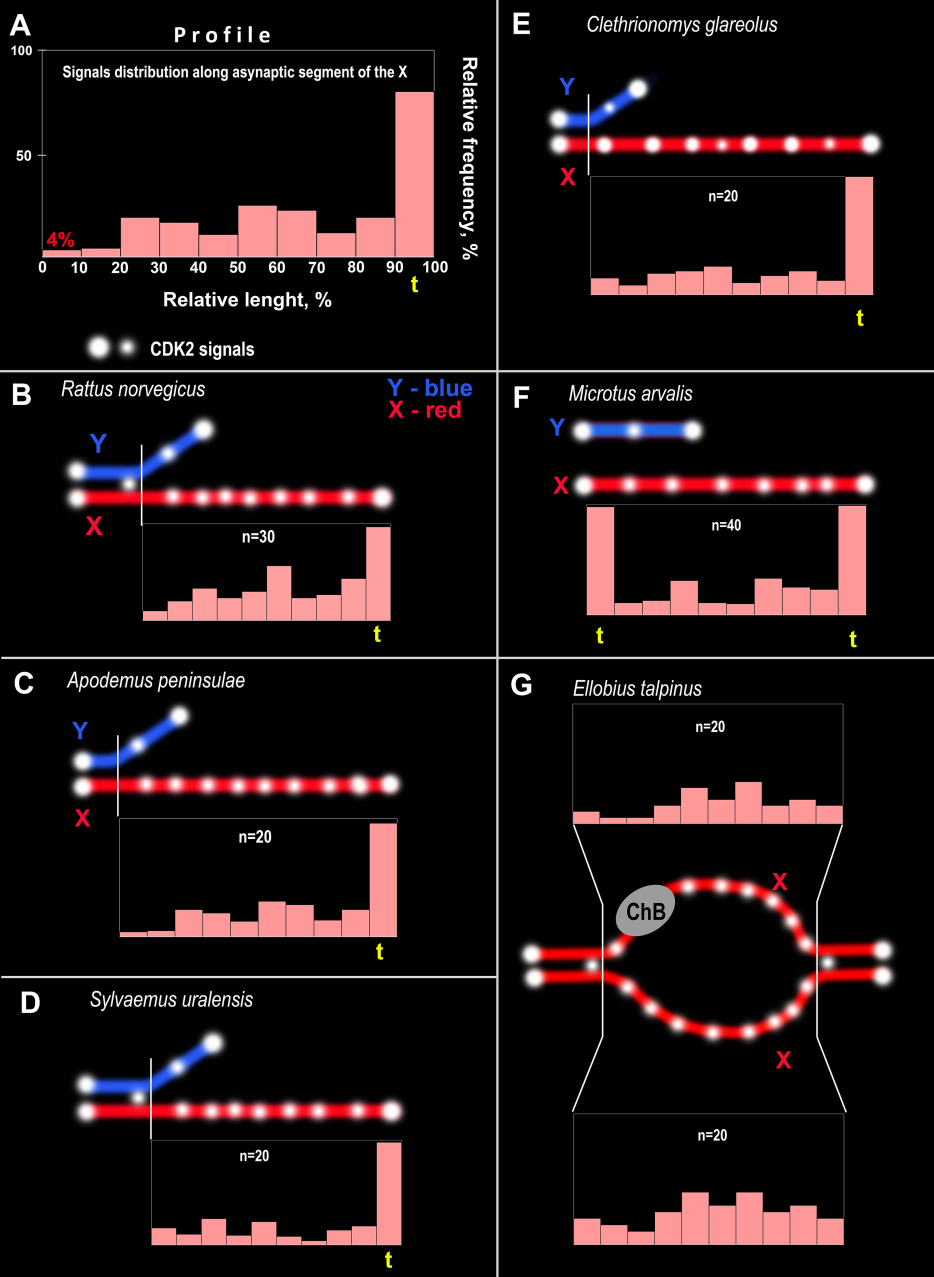
CDK2 immuno-profiles of the asynaptic segments of the X chromosomes and schemes of CDK2 signals in the rodent pachytene sex bivalents. The yellow <<t>> indicates the telomeric segment of the meiotic chromosome. ChB – chromatin dense body of mole vole X chromosome. (A) Schematic explaining the CDK2 profiles. The length of the X chromosome was divided into 10 parts and CDK2 signals were identified in each part; their frequencies are reflected by the height of the light pink bars, *i.e.,* the higher the bar, the more frequently the signals were found in this chromosome region. CDK2 profiles for: (B) *R. norvegicus*, (C) *A. peninsulae*, (D) *S. uralensis*, (E) *C. glareolus*, (F) *M. arvalis*, and (G) *E. talpinus*.

CDK2 signals could be located with different frequencies at each site of the asynaptic segment of the X chromosome. The CDK2 distribution along the X chromosomes varied significantly for all species studied. Graphically, the CDK2 distribution was presented in immuno-profiles (Fig. 4A). Analysis of these profiles allowed us to determine that CDK2 signals usually (with rare exceptions) lay within the telomeric regions (the highest bar on the profiles, see Figs. 4B–4F) and had an irregular localization along the asynaptic segments of the X chromosome (Figs. 4B–4G) in all rodents. In our previous work, we presented micro photos of *E. talpinus* meiotic chromosomes stained by antibodies against SYCP3/CDK2 (Matveevsky et al., 2021). Here, since the male mole vole sex bivalent is represented by two X chromosomes, we present two CDK2 profiles of which a chromatin dense body (ChB) was previously found on one (Kolomiets et al., 1991; Kolomiets, Matveevsky & Bakloushinskaya, 2010; Matveevsky, Bakloushinskaya & Kolomiets, 2016). There are no CDK2 signals within the ChB, therefore very short bars were observed on the profile (ChB position was variable) (top diagram in Fig. 4E).

### Domain organization and other parameters of BRCA1

The conservation of the BRCA1 enzyme was also investigated. In a previous study using a narrow set of species (Arabidopsis and some vertebrates), we showed low conservation of this protein even within vertebrates (Grishaeva, 2018). Over the years, annotated protein orthologs in other plant and invertebrate species have appeared in databases. Here, we first analyzed the conservation of BRCA1 in eight distant species. The sizes of the orthologs varied greatly, from 612 aa in nematodes up to 2,942 aa in the owl limpet. All of the orthologs contained similar domains only at their protein ends (two C-end domains and an N-end RING finger, which is also typical for this protein). The single domains in the middle of the molecule varied among samples. Additionally, numerous active sites have been identified in some orthologs. The pIs of the proteins were all in a narrow interval (5.15–6.79).

Next, we analyzed mammalian BRCA1 orthologs in species including humans and rodents. In the database, the orthologs were in two groups, full-length (human, mice, rat, mole rat, and hamster) and partial (shrew, vole, field mouse, and mole voles). The domain structures of the 6 full-length proteins were very similar; the domains at both ends of the proteins were identical, as were the active sites. In the middle part of the protein, a serine-rich BRCA1 domain was detected. Fragmented (partial) proteins had 1–2 domains only. The pIs of the full-length proteins were in a narrow interval (5.15–5.85), while the shortened proteins had different pIs (7.23–8.87); therefore, there were two different groups of orthologs concerning this parameter. The secondary structure of proteins was more diverse; partial proteins did not have alpha-helical regions and, it must be noted that any functional domains are lacking in the alpha-helical regions.

### Conserved amino acid motifs in BRCA1 proteins

**Figure 5:**
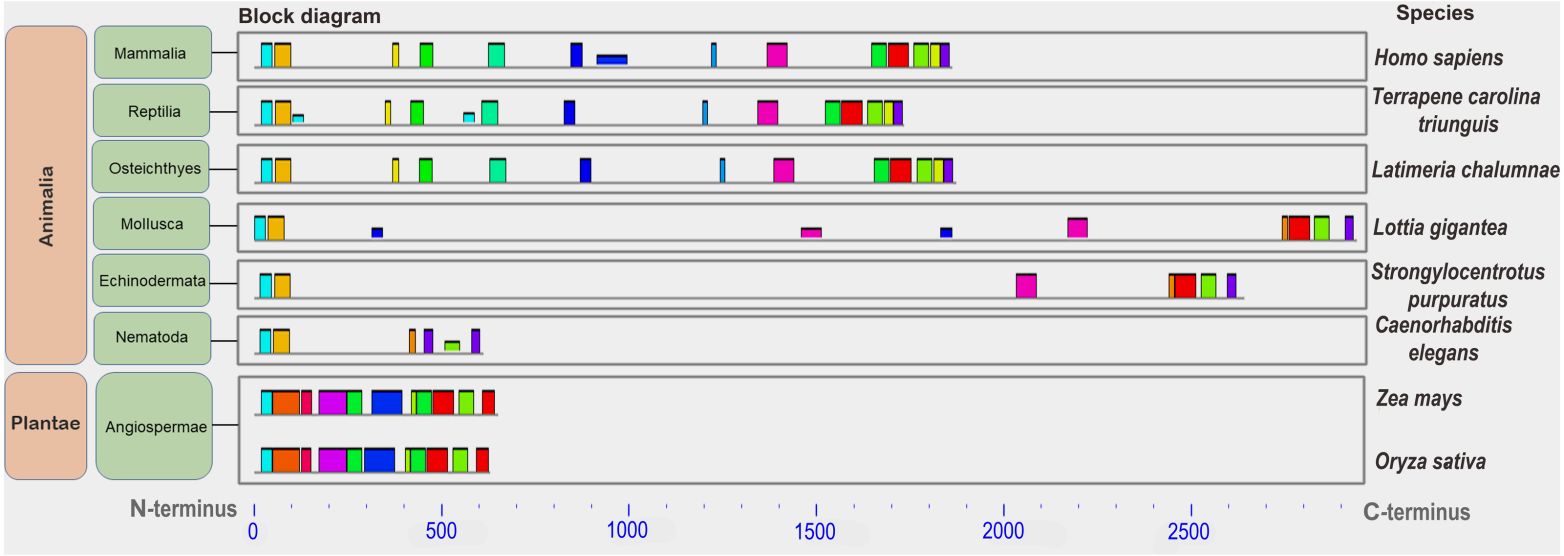
A set of conserved amino acid motifs in BRCA1 proteins of different eukaryotes. Human (Hs) (mammals); turtle (Tct) (reptiles); coelacanth (Lch) (bony fish); owl limpet (Lg) (mollusks); purple sea urchin (Spu) (echinoderms); roundworm (Ce) (nematodes); maize (Zm), rice (Os) (plants). Identical motifs are indicated by rectangles of the same color and size. Distant taxa have different architecture of amino acid motifs.

Low conservation of the BRCA1 orthologs can be deduced from the great variation in protein lengths—from 612 aa to 2,942 aa. Analysis of the set of conserved amino acids in the orthologs of large eukaryote taxa (plants and animals) showed that similar motifs were detected only at the ends of proteins carrying functional domains (Fig. 5). Plant proteins were similar only to one another, but not to animal orthologs. In vertebrates, single small motifs were similar (in addition to the similar terminal motifs of all orthologs).

**Figure 6:**
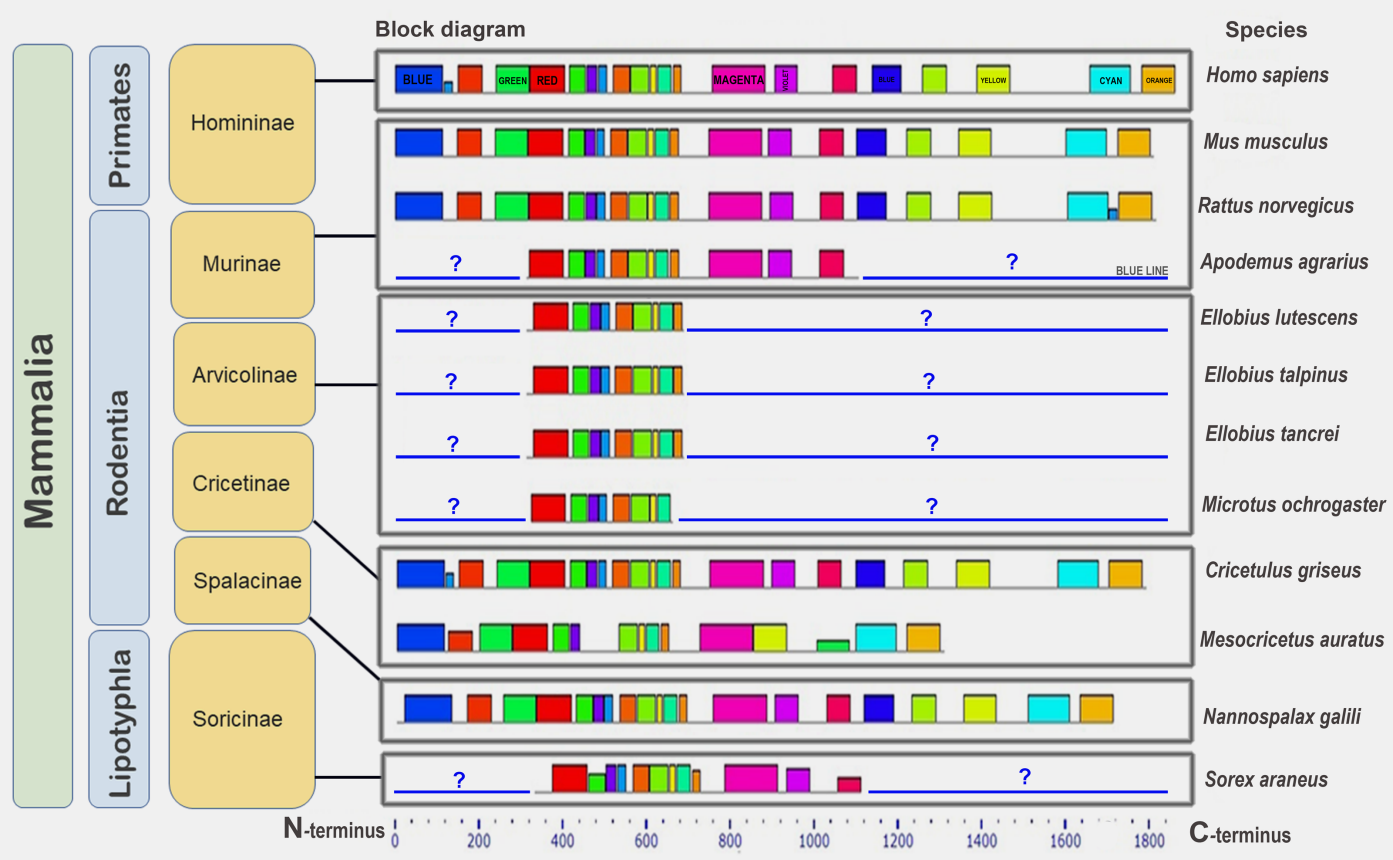
A set of conserved amino acid motifs in BRCA1 proteins of mammals. Human (*H. sapiens*) (primates); mouse (*M. musculus*), rat (*R. norvegicus*), vole (*M. ochrogaster*), mole rat (*N. galili*), Chinese (*C. griseus*) and golden (*M. auratus*) hamsters, field mouse (*Apodemus agrarius*), mole voles (*E. lutescens*, *E. talpinus*, *E. tancrei*) (rodents); and shrew (*S. araneus*) (insectivores). Identical motifs are indicated by rectangles of the same color and size. Blue question mark and blue line represent unknown regions in partially annotated proteins.

Next, we studied BRCA1 orthologs in vertebrate species (mammals, birds, and amphibians). Lower conservation of the BRCA1 protein compared to that of CDK2 was confirmed (see Fig. S5). The set of motifs was individual to every ortholog group. End motifs of proteins were similar. Finally, we studied the conservation of BRCA1 orthologs from 12 mammalian species. The set of conserved amino acid motifs was identical with minimal differences (Fig. 6).

The golden hamster (Ma) BRCA1 apparently carries several deletions in the middle portion of the molecule and, therefore, lacks some motifs. Short (partial) proteins have sets of motifs identical to the corresponding regions of the full-length proteins (Fig. 6). Thus, the full-length orthologs of the BRCA1 protein are very similar among mammals.

### Immunocytological analysis of BRCA1 localization in spermatocytes

**Figure 7:**
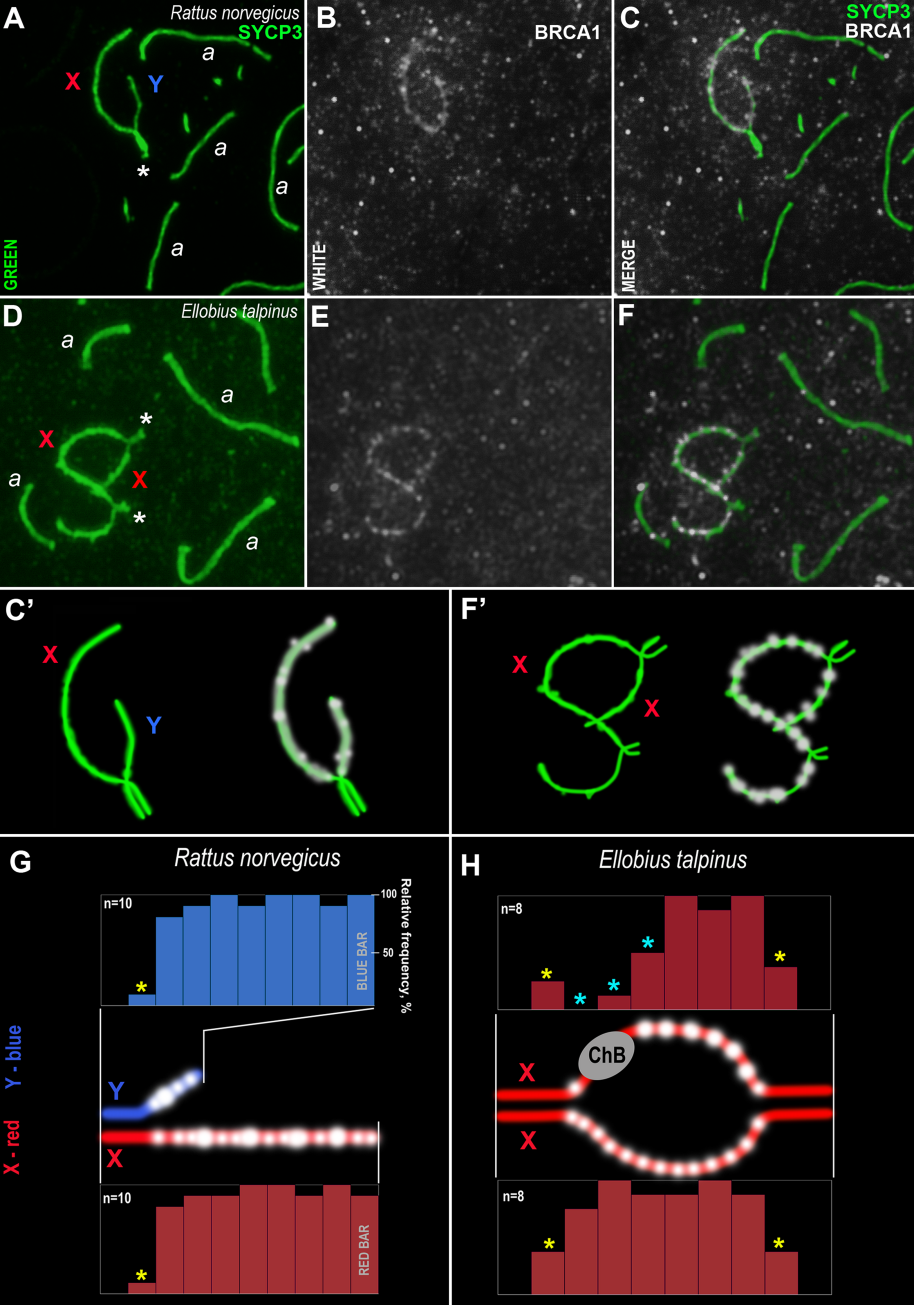
Rodent pachytene spermatocytes and BRCA1 immuno-profiles of sex bivalents. Synaptonemal complexes (SCs) were immunostained using antibodies to SYCP3 protein (green) and BRCA1 using antibodies to BRCA1 (white). Portions of spermatocytes with autosomal SCs (designated as *a*) and sex bivalents are present in *R. norvegicus* (A–C, C’) and *E. talpinus* (D–F, F’). White asterisks indicate synaptic regions of sex chromosomes (A, D). The female sex chromosome is marked with a red “X”. The male sex chromosome is marked with a blue “Y”. BRCA1 profiles reflect the distribution of BRCA1 signals along the sex chromosomes (blue bars for the Y, red bars for the X chromosomes; G, H). Short bars (yellow asterisks) reflect signals in those sites where the border between the synaptic and asynaptic regions was varied. The sizes and localization of ChBs were variable; therefore, short bars in these segments are presented (light blue stars in the top profile in H).

An analysis of the BRCA1 distribution in rat and mole vole mid pachytene spermatocytes was performed. As a rule, BRCA1 was not identified within autosomal SCs or rare immuno-dots could be seen (Fig. 7). In the sex bivalents of these rodents, numerous BRCA1 dots were localized along the asynaptic regions, while the BRCA1 foci were absent in the synaptic regions (Figs. 7A–7C, 7C’, 7D–7F, 7F’). It should be noted that the specific BRCA1 staining was irregular; specific immuno-signals could be detected in some nuclei while, in others on the same slide, there were only nonspecific foci in the nucleoplasm (sometimes in all nuclei on one slide). These findings were typical for both species of rodents. In this regard, we used BRCA1 antibodies with a weak dilution (1:20), however, a high level of nonspecific binding was detected (smearing or dirty effect). Since the sex chromosomes in male turkeys and frogs are homomorphic (ZZ), we analyzed BRCA1 staining in zygotene spermatocytes as BRCA1 is located in unpaired chromosome regions (Turner et al., 2005). However, specific BRCA1 foci were not detected in either the asynaptic or synaptic regions (Fig. S8).

Analysis of the BRCA1 profiles of the two rodents revealed common patterns: numerous BRCA1 dots were located next to each other in the unpaired axes of both the X and Y chromosomes (Figs. 7G–7H). No BRCA1 foci were present in the synaptic regions.

## Discussion

The evolution of living systems occurs at different biological levels, from the biosphere and ecosystems to cells and molecules. Many fundamental cell processes show signs of conservation. For example, programmed cell death (Yuan, 1996), microtubule-capture mechanisms (Gundersen, 2002), cell division, and meiosis (Bogdanov, 2003; Egel & Penny, 2007). These processes are based on the functioning of hundreds or thousands of proteins and complex protein compounds. Proteins are known to have different evolutionary rates: some proteins are substantially rearranged in a short time, while others are conserved without changes for a long time (Wilson, Carlson & White, 1977). What could be the rationale for such conservation? According to one of the hypotheses (“functional importance”, Kimura & Ohta, 1974), the most crucial molecules or parts of them exhibit lower evolutionary rates. Some studies, however, have found a weak correlation between the functional significance and evolution rate of proteins (see review Zhang & Yang, 2015). The recent development of genomic and bioinformatics modern methods made possible to assess the homology of nucleotide and amino acid sequences and to conclude that they are conserved.

The evolution of proteins can be accompanied by changes not only to their amino acid sequences but also their structural and spatial molecular organization (Ingles-Prieto et al., 2013), although several limiting factors exist (Lipman et al., 2002). Nevertheless, the question remains why some proteins are highly evolutionary conserved while others undergo significant modifications (Kisters-Woike, Vangierdegom & Müller-Hill, 2000). Both trends are traced in the evolution of proteins involved in meiosis (Bogdanov, Grishaeva & Dadashev, 2007). In some cases, proteins are that not homologous to each other at all are involved in the construction of analogous cell structures. For example, the proteins of the SC’s CEs (nonhomologous proteins: SCP1 in mammals, Zip1 in yeast, and ZYP1 in Arabidopsis) have a similar structure consisting of three amino acid domains, including a central one (α-helix capable of forming a second-order helix, supercoiling) and two terminal domains of the globule type (Bogdanov, 2008). The analogy of house construction has been used to describe such a situation, in which the same elements (walls, roof, windows, *etc.*) can consist of different materials (Bogdanov & Kolomiets, 2007). It follows from this that those unrelated proteins in distant taxa that form the same intracellular structures are an example of convergent evolution (Gough, 2005; Forslund, Kaduk & Sonnhammer, 2012; Gilson et al., 2017). Comparison of the protein conservation within different taxonomic groups (multifunctional, such as CDK2 and BRCA1, or highly specific) allows us to clarify their specificity and the direction of evolution.

### CDK2 confirmed as a conserved protein bioinformatically and immunocytochemically

CDK2 is a multifunctional kinase, active not only in the cell cycle (Morgan, 1997) but also in various processes during germ cell development (Palmer, Talib & Kaldis, 2019 and refs therein). The last 20 years have been marked by active accumulation of data regarding the role of CDK2 in meiosis. It is now generally accepted that the synthesis and degradation of CDKs are necessary for meiotic progression, including prophase I (Morelli & Cohen, 2005; Cohen, Pollack & Pollard, 2006). In this context, the previous research results can be summarized in four main points:

1. CDK2 is involved in the attachment of telomeres to the nuclear lamina (Viera et al., 2015; Mikolcevic et al., 2016).

2. CDK2 plays an important role in chromosome synapsis and SC formation (Ortega et al., 2003; Viera et al., 2009).

3. CDK2 is required for accurate meiotic recombination and chiasma formation (Ward et al., 2007; Viera et al., 2009; Palmer et al., 2020).

4. CDK2 is involved in the correct formation of the sex body in spermatocytes (Viera et al., 2009).

We showed that CDK2 is a highly conserved kinase in different eukaryotic lines (fungi, plants, vertebrates, and invertebrates) (Fig. 1). This is evidenced by a similar set of conserved amino acid motifs in the kinase orthologs studied. The phylogenetic tree (Fig. S9) of the same ortholog set as in Fig. 1 also shows short evolutionary distances between proteins and their affiliation to the corresponding lines of Eukaryotes. Only CDK2 of Nematode *C. elegans* is situated away from other proteins, especially far from that of Invertebrate *D. melanogaster*. Additionally, the pIs of the proteins were close among the vertebrates. Work published by Nuñez Hernandez et al. (2019) comparing shrimp CDK2 with orthologs in other organisms confirms our results. Moreover, the set of conserved motifs was similar among humans, rodents and insectivore studied (Fig. S4).

Immunocytochemical research has revealed a conserved CDK2 pattern for rodent meiotic chromosomes. Thus, CDK2 always colocalizes with the marker of late nodules of recombination, the MLH1 protein (Ashley, Walpita & De Rooij, 2001), and is also always immuno-identified in the telomeric regions of chromosomes (Viera et al., 2015; Mikolcevic et al., 2016). These data were confirmed by us earlier in studies on seven rodent species (Matveevsky et al., 2021), with three additional rodent species added in the present study. However, the absence of CDK2 foci in the telomeric ends of all frog meiotic chromosomes, as well as in many of the turkey meiotic chromosomes, suggests that this kinase may not be involved in telomeric attachment to the nuclear envelope in these species. Undoubtedly, this assumption should be investigated further.

It should be noted that, in the present study, a variable CDK2 pattern was observed for the asynaptic regions of the X and Y chromosomes at the mid pachytene stage, similar to the results of Matveevsky et al. (2021). Thus, numerous CDK2 signals were observed along the rodent X chromosomes making it possible to construct “kinase” diagrams (Fig. 4). The confirmed role of this enzyme in the unpaired axes of sex bivalents has not currently been established. However, it has been hypothesized that CDK2, along with other proteins, may play a role in the system of meiotic checkpoints (Ward et al., 2007; Mikolcevic et al., 2016), for example through interactions with MSCI proteins, as we have previously suggested (Matveevsky et al., 2021). However, these data must be experimentally proven.

The flexibility of CDK2 is also an evolutionarily conserved trait (Bártová, Koča & Otyepka, 2008). However, CDK-based gene tree allows the isolation of large taxa: vertebrates, insects, protozoa, and fungi (Riley & Krieger, 1995). Interestingly, all unicellular eukaryotes, including yeasts, have the amino acids Ala, Val, and Asp/Glu in CDK2 at positions 21, 83, 88, and 116, while multicellular eukaryotes have Gly, Ile, Ser, and Ile at these respective positions (Riley & Krieger, 1995). It is assumed that such amino acid replacements may increase the versatility of the CDK2 molecule in performing multiple functions. Minimal differences in motifs were noted at the C-terminus of CDK2 in *D. melanogaster* and *C. elegans* (Fig. 1). How can such high conservation of this protein be explained? First of all, CDK2 participates in many cellular processes and interacts with numerous proteins. On the other hand, CDK2 is a small protein that is almost entirely represented by one functional domain, *i.e.,* the entirety of the molecule is essential in performing its numerous functions.

Bioinformatics data on CDK2 conservation suggests the possibility of using commercial antibodies for research in a lot of species. Studies of CDK2 in the meiotic chromosomes of other vertebrates will be relevant.

### BRCA1 as an evolutionary dynamic protein with low conservation

In our previous work, we have shown that, in some models, BRCA1 orthologs are similar in the terminal regions of the protein only, among vertebrates (Grishaeva, 2018). In the present work, we examined BRCA1 conservation in three steps. The first step included representatives of distant taxa (plants, vertebrates, and invertebrates). Significant similarities were found only in two plant representatives, specifically maize and rice. All studied orthologs had small common motifs at the N- and C-termini of the molecules, and there were fewer such motifs in plants (Fig. 5). In vertebrates, small motifs in the middle part of the molecule were also similar. The secondary structure of orthologs was diverse (not conserved), however, the pIs of the proteins were quite similar, despite the differences in amino acid sequences. In general, our previous conclusion regarding low BRCA1 conservation in the eukaryotes was confirmed *via* many of the model species (Grishaeva, 2018). The phylogenetic tree for these orthologs (Fig. S10) shows longer evolutionary distances between proteins in comparison with orthologs of CDK2 (see Fig. S9). In spite of this fact, their affiliation to the corresponding lines of Eukaryotes is present.

The second step of the analysis involved comparing BRCA1 conservation within only vertebrates (Fig. S5), similar to that done for CDK2. Common motifs for all orthologs were found only at the ends of the molecule, as in the first step (Fig. 5). Each of the three taxa demonstrated both common and different motifs. Immunocytochemically, we revealed BRCA1 staining specificity only for rodents. Thus, low BRCA1 conservation was confirmed for vertebrates. This means that commercial antibodies against specific regions of human or mouse BRCA1 may not be suitable for use in distant organisms.

In the third step of the analysis, BRCA1 conservation was studied within mammals, including humans, rodents, and insectivores. Orthologs were found to be quite similar in terms of pIs in both full-length and fragmentary proteins, although they differed in secondary structure. The set of conserved amino acid motifs was almost identical in all the full-length proteins, except the reduced BRCA1 of the golden hamster (Fig. 6).

Since we do not yet know which sequences are located at the N- and C-termini of partially annotated proteins (for example, mole vole proteins), conclusions about their conservation can only be drawn from the fragments available. These parts of the molecules are very similar to the corresponding parts of full-length proteins (Fig. 6).

BRCA1 is a multifunctional protein, not only in somatic cells (Marcon & Moens, 2005; Deng, 2006), where it is also involved in interactions with CDK2 (Hydbring, Malumbres & Sicinski, 2016), but also in various processes occurring during meiosis, “through at least two different mechanisms, the recruitment of DNA damage-repair proteins to sites of DNA damage, and the regulation of the expression of DNA damage repair genes” (Xu et al., 2003). Based on previous research, the main meiotic functions of BRCA1 can be summarized as follows:

1. BRCA1 finds unpaired chromosome regions (Scully et al., 1997) and then recruits ATR in the AEs of the sex bivalent and autosomes, thereby initiating MSCI (Turner et al., 2004; Marcon & Moens, 2005) and MSUC (Turner et al., 2005).

2. BRCA1 is necessary for proper formation of pericentric heterochromatin and correct compartmentalization of the sex bodies in meiotic prophase I (Broering et al., 2014).

3. BRCA1 may be critical for the correct progression of meiotic recombination, at least for *C. elegans* (Li et al., 2018; Janisiw et al., 2018). The absence of BRCA1 in mutant male mice had a moderate effect on meiotic recombination, while in mutant females it did not affect this process at all (Broering et al., 2014).

An immunocytological study on rat and mole vole meiocytes showed that the antibodies produced against the human BRCA1 protein were specific for some chromosomes of both rodents. Numerous BRCA1 foci were located in a similar pattern on the unpaired regions of the rat and the mole vole sex chromosomes (Fig. 7), which indicates the initiation of the MSCI process (for the mole vole, see Matveevsky, Bakloushinskaya & Kolomiets, 2016). The distribution of BRCA1 (as well as CDK2) had differences for the two Ellobius X chromosomes, due to the presence of ChBs. These features are part of the unique epigenetic landscape of the male mole vole XX body (Matveevsky, Bakloushinskaya & Kolomiets, 2016; Gil-Fernández et al., 2021). Irregular staining of meiotic chromosomes using antibodies to BRCA1 within the same slide may be due to the fact that antibodies were obtained not against a linear fragment of the molecule, but against an epitope, *i.e.,* combinations of surface regions of the N-terminal fragment of the protein with a certain folding. In this case, in some nuclei, this epitope may be broken due to insufficiently accurate folding of protein fragments and become “invisible” for given antibody. This possibly depends on the pachytene substage and/or on fluctuations in the conditions and fixation of nuclei spreading, as well as singularities of antibody affinity. Additionally, it is not yet clear what the boundaries and lengths of the full-length copies of the mole vole BRCA1 are, as only partial data on amino acid sequences are currently known. If their N-terminal fragments do not coincide with that of the human protein, it becomes clear why antibodies developed against the human BRCA1 epitope (the first 304 aa) irregularly stain the mole vole meiotic chromosomes. Current known Motif 1 (the first common motif among humans and mole voles, Fig. 6) begins after approximately the 300th human amino acid.

## Conclusions

Protein molecules, depending on their size, length, complexity of an organization, and functions may evolve at various rates (Agozzino & Dill, 2018). It should be noted that various protein domains can be subjected to evolutionary pressure, with some being conserved by stabilizing selection while others are made variable by directional selection (Björklund et al., 2005; Buljan & Bateman, 2009). In this work, we found two enzymes (CDK2 and BRCA1) with a wide range of functions that work side by side in some cellular processes possess different levels of conservation, which is likely associated with various dynamics and the duration of their evolutions. It is estimated that BRCA1 genes originated approximately 1.6 billion years ago, before the separation of the two kingdoms of plants and animals (Pfeffer, Ho & Singh, 2017). After this point, BRCA1 underwent significant reorganization, such as the emergence of a plant-specific PHD domain. Even though a significant number of conserved amino acid motifs of BRCA1 remain between mice and humans (Grishaeva, 2018), the identity of these vertebrate BRCA1 homologs is only 56% (Pfeffer, Ho & Singh, 2017). The presence of CDK2 in the three kingdoms (fungi, plants, and animals) implies that this protein also originated about 1.6 billion years ago, and possibly earlier. Clearly, both enzymes are ancient and have traversed a huge evolutionary path. However, given their multifunctionality, the two proteins have had different evolutionary strategies: (1) preservation of the architecture of conserved amino acid motifs in close and distant taxa (*e.g.*, CDK2) or (2) significant reorganization/reconstruction of amino acid motifs with an extremely low level of conservation (*e.g.*, BRCA1). The phenomenal range of the quantity and quality of amino acids in the molecules, their temporal and spatial organization, the formation of homologs, orthologs, and paralogs, as well as the time of origin, underlie the extraordinary diversity of proteins and serve as a manifestation of the mosaic evolution of biological systems.

##  Supplemental Information

10.7717/peerj.12231/supp-1 Supplemental Information 1 Supplemental Figures

## Additional Information and Declarations

### Data Availability

The following information was supplied regarding data availability: The data is available at Mendeley: - Matveevsky, Sergey (2021), “CDK2 and BRCA1 conservation (Immunostaining. Bioinformatics)”, Mendeley Data, V1, doi: https://dx.doi.org/10.17632/whpxpxprhd.1, - Matveevsky, Sergey (2021), “Raw data (qualitative analysis: CDK2-, BRCA1-immunoprofiles)”, Mendeley Data, V1, https://dx.doi.org/10.17632/xzjrwp8fws.1.
