# Supplementary material for: Heterogeneity in conservation of multifunctional partner enzymes with meiotic importance, CDK2 kinase and BRCA1 ubiquitin ligase
Source: PeerJ. 2021 Sep 27;9:e12231. doi: 10.7717/peerj.12231 (PMC8483008; doi:10.7717/peerj.12231)
Supplement: Supplemental Information 1 [file peerj-09-12231-s001.doc]

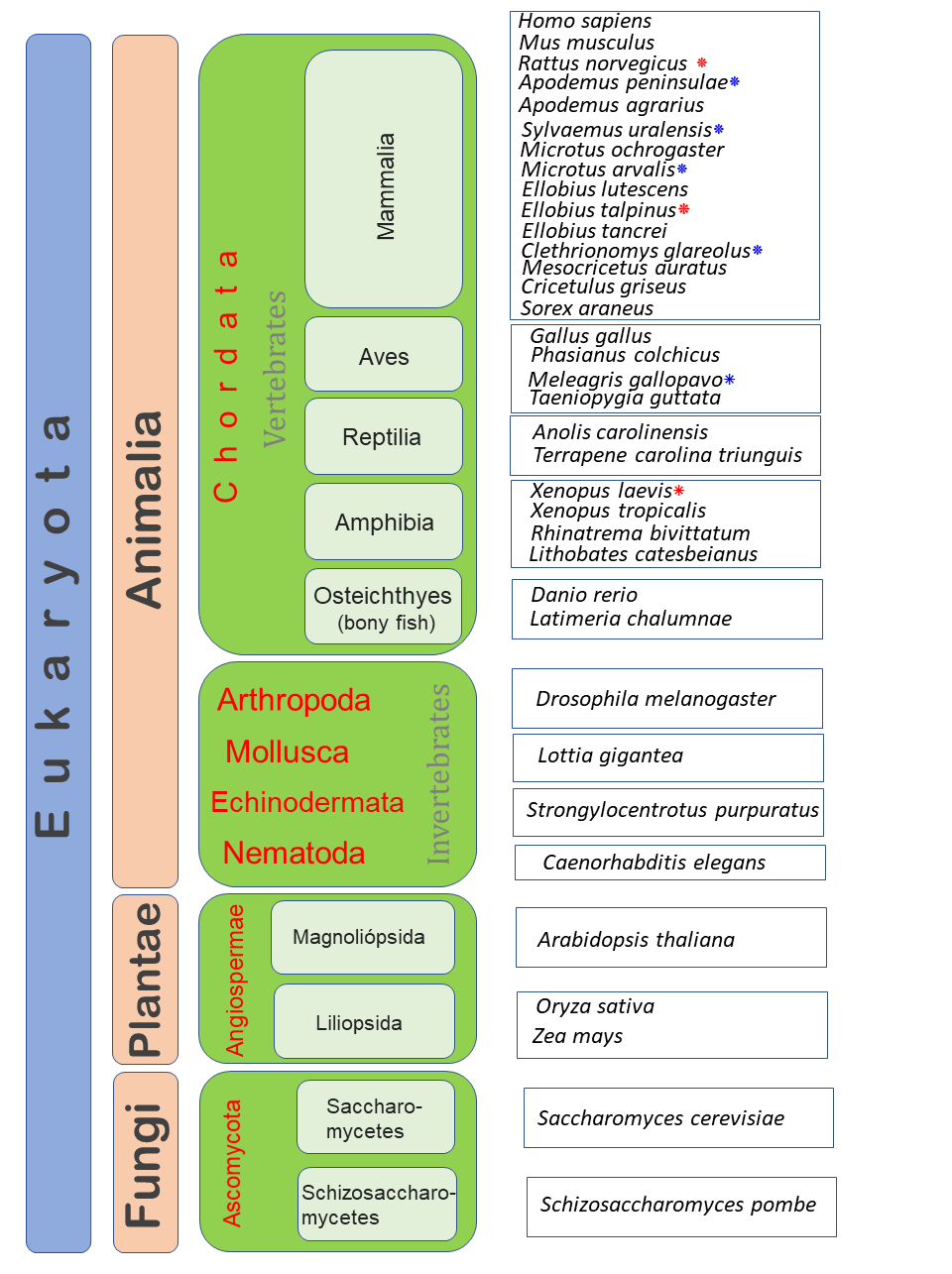


**Figure S1.** Research objects and their taxonomic position. A red asterisk denotes research by bioinformatics and immunocytochemistry methods; a blue asterisk - immunocytochemically; without an asterisk - bioinformatically.


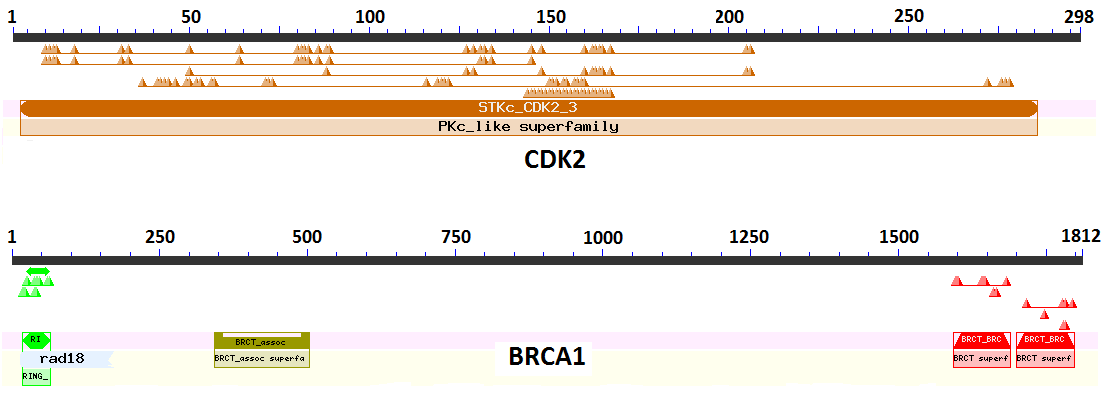


**Figure S2.** Conserved functional domains in CDK2 and BRCA1 of *Mus musculus*. Upper half:

domain STKc_CDK2_3 (serine/threonine kinase, cyclin-dependent kinase 2 and 3 from the

PKc_like superfamily, brown rectangle) and numerous active sites (brown triangles) in the

CDK2 molecule. Down half: coinciding domains Ring_finger (green rectangle) and rad18, BRCT_associated (gray rectangle), two BRCT_BRC domains (red rectangles) and some active sites (green triangles) in the BRCA1 molecule. The order of amino acids is shown (black thick line). Other explanations are in the text.


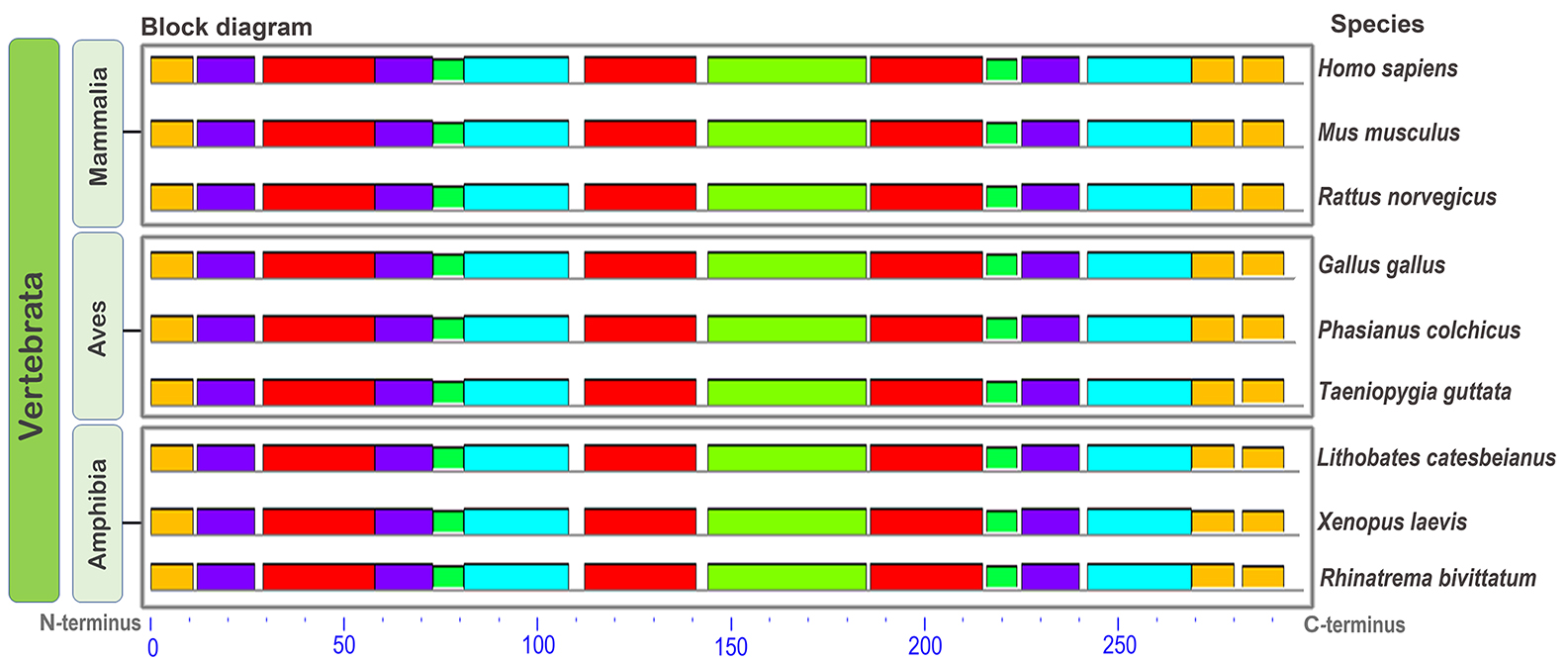


**Figure S3.** A set of conserved amino acid motifs in CDK2 proteins of only vertebrates: human (*Homo sapiens*), mouse (*Mus musculus*), rat (*Rattus norvegicus*) (mammals); chicken (*Gallus gallus*), zebra finch (*Taeniopygia guttata*), pheasant (*Phasianus colchicus*) (birds); caecilian (*Rhinatrema bivittatum*), bullfrog (*Lithobates catesbeianus*) and clawed frogs (*Xenopus laevis*) (amphibians). Identical motifs are indicated by rectangles of the same color and size. The N and C termini of proteins are indicated. A motif set is identical for all studied species.


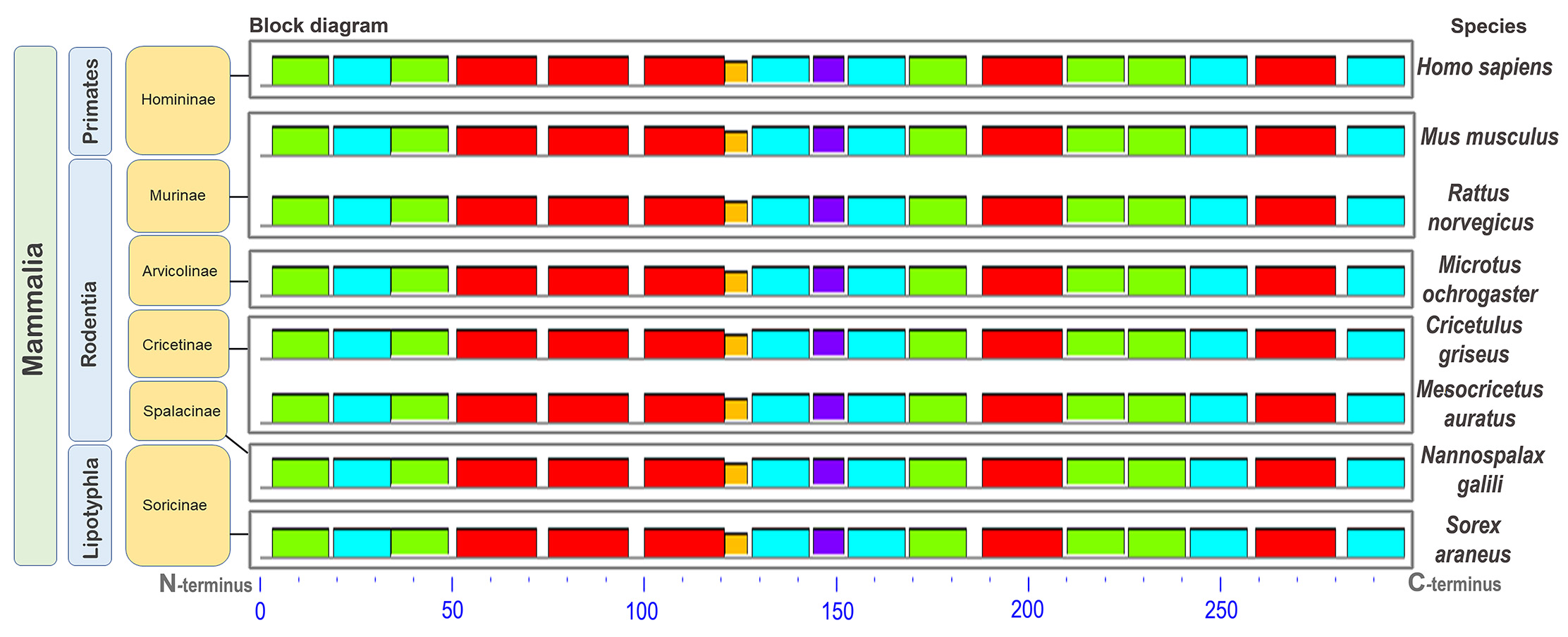


**Figure S4.** A set of conserved amino acid motifs in CDK2 proteins of mammals representing a narrow group of vertebrates: human (*Homo sapiens*), mouse (*Mus musculus*), rat (*Rattus norvegicus*), vole (*Microtus ochrogaster*), mole rat (*Nannospalax galili*), shrew (*Sorex araneus*), hamsters (*Cricetulus griseus*, *Mesocricetus auratus*). Other explanations are as in figure S3. A motif set is also identical for all studied species.


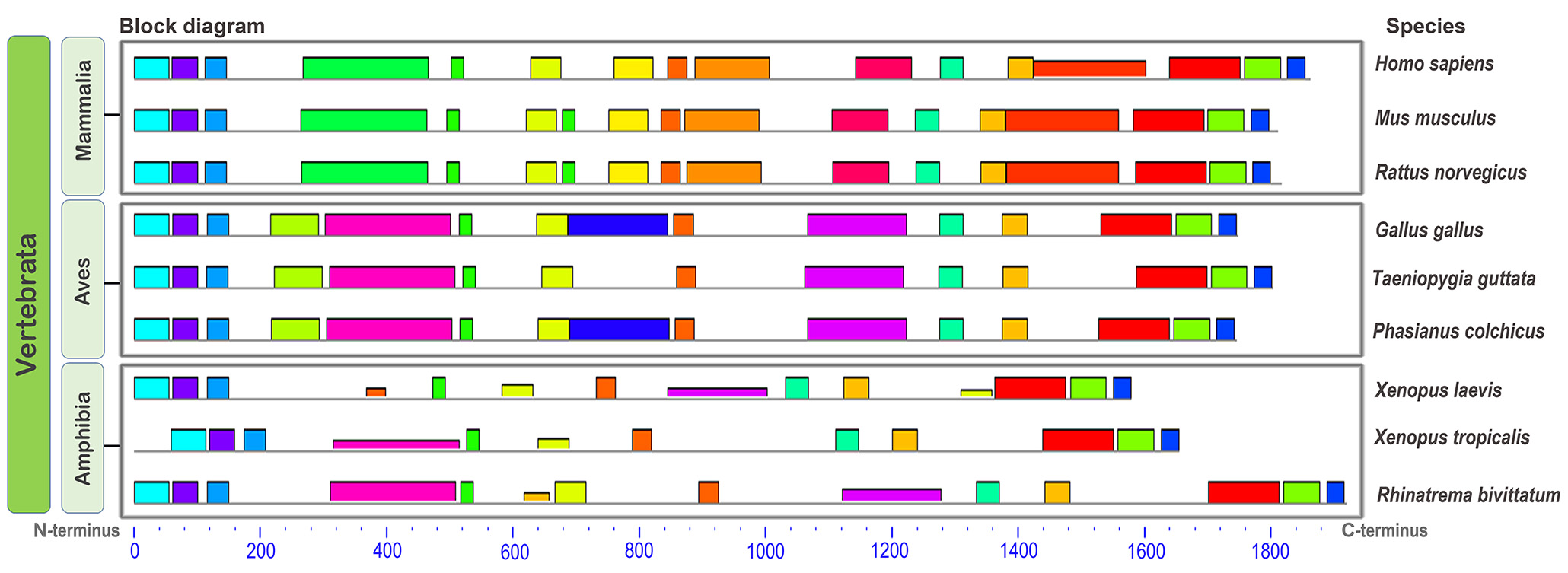


**Figure S5.** A set of conserved amino acid motifs in BRCA1 proteins of vertebrates: human (*Homo sapiens*), mouse (*Mus musculus*), rat (*Rattus norvegicus*) (mammals); chicken (*Gallus gallus*), zebra finch (*Taeniopygia guttata*), pheasant (*Phasianus colchicus*) (birds); clawed frogs (*Xenopus laevis*, *Xenopus tropicalis*) and caecilian (*Rhinatrema bivittatum*) (amphibians). Other explanations are as in figure S3. Motifs sets are similar in proteins inside the groups of Mammals and Aves and, in less extent, in Amphibia. But similarity between groups of species is restricted only by terminal motifs.


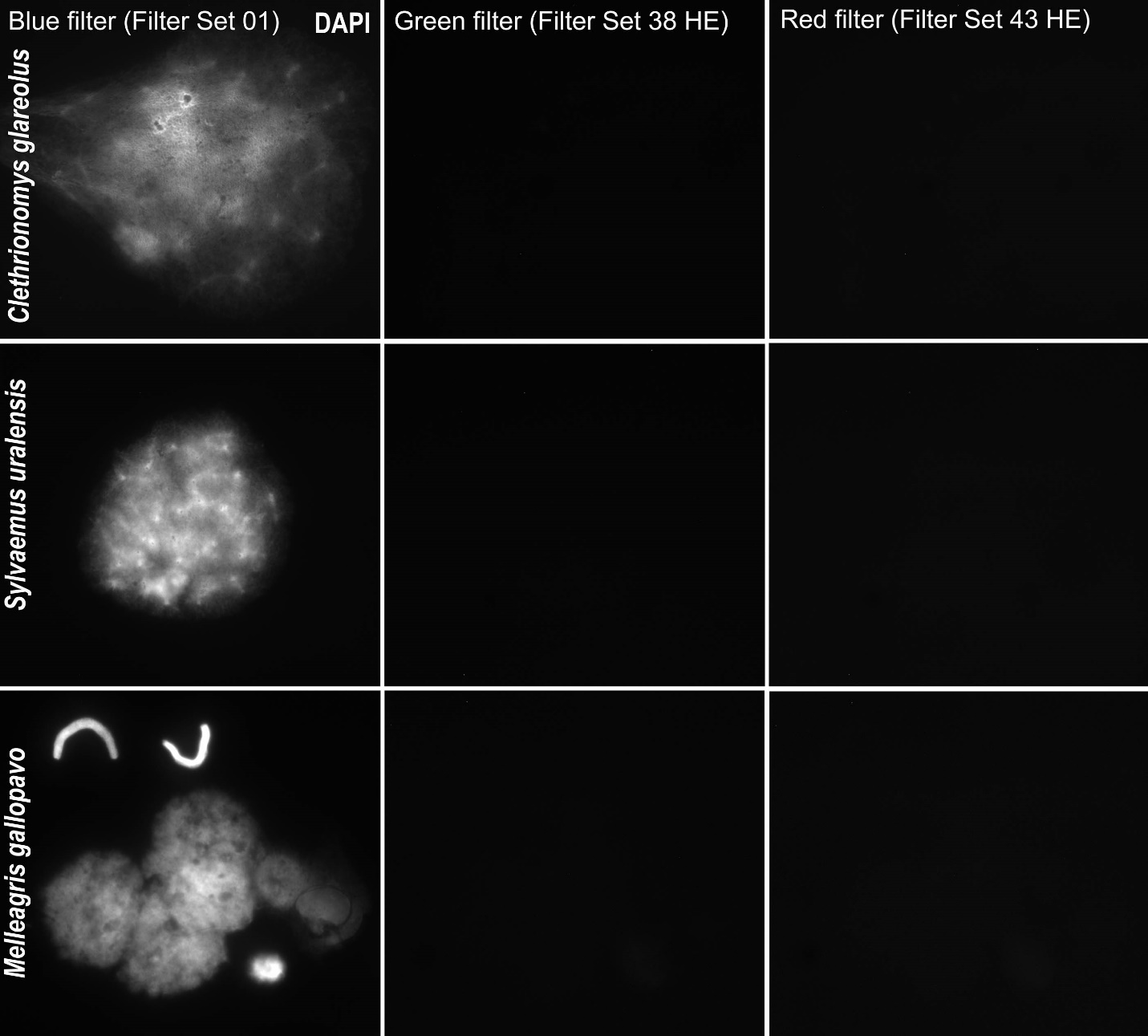


**Figure S6.** Negative control of immunostaining: overnight incubation with antibody dilution buffer (ADB) and without primary and secondary antibodies for three species. To identify cells, the slides were stained with DAPI (Filter set 01, left column). At standard exposure, there were no immuno-signals on the green (Filter set 38HE, central column) and red (Filter set 43HE, right column) filters.


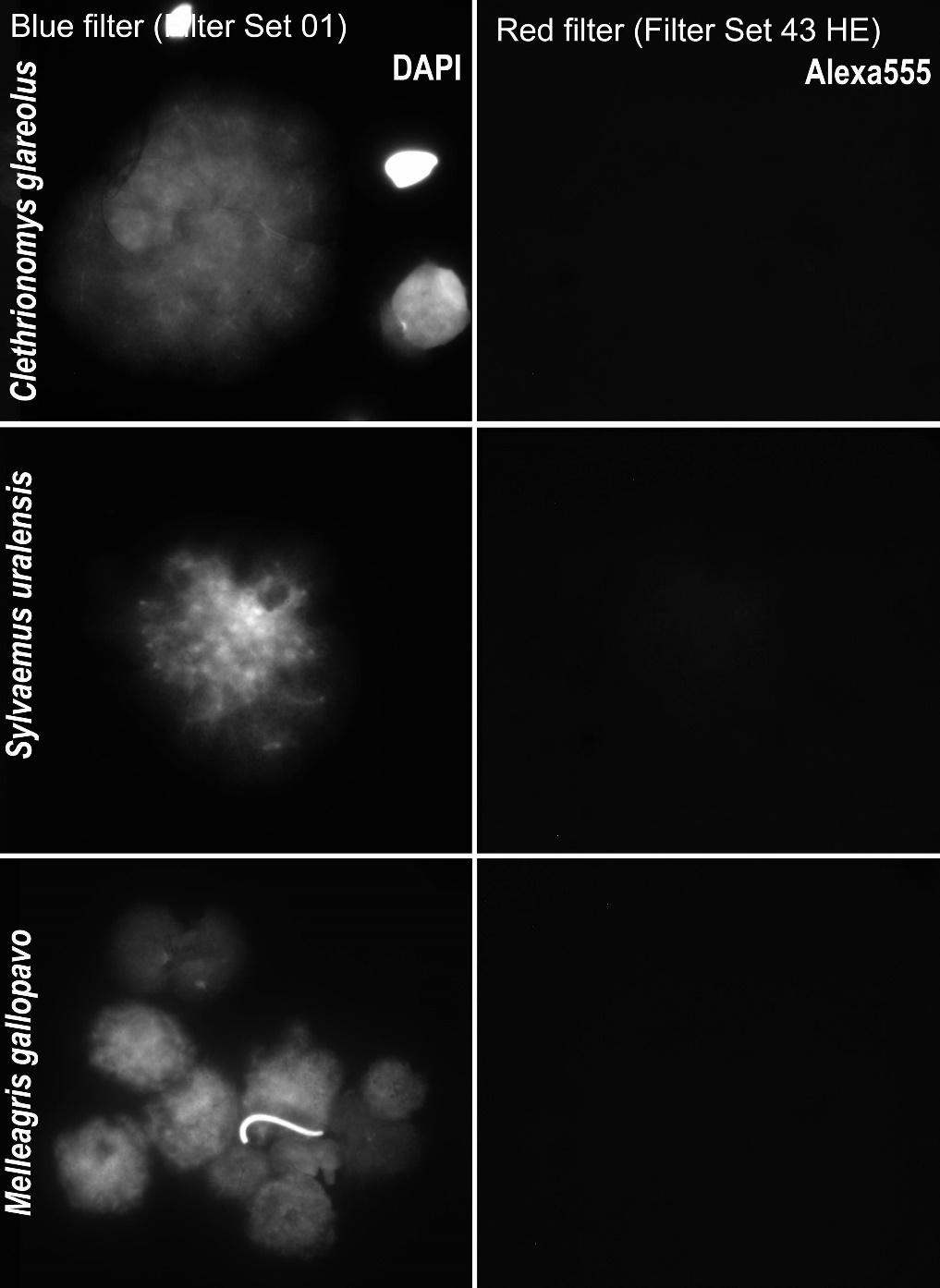


**Figure S7.** Negative control of immunostaining: overnight incubation with secondary goat anti-mouse IgG Alexa Fluor 555-conjugated antibody for three species. To identify cells, the slides were stained with DAPI (Filter set 01, left column). At standard exposure, there were no immuno-signals on the red (Filter set 43HE, right column) filter.


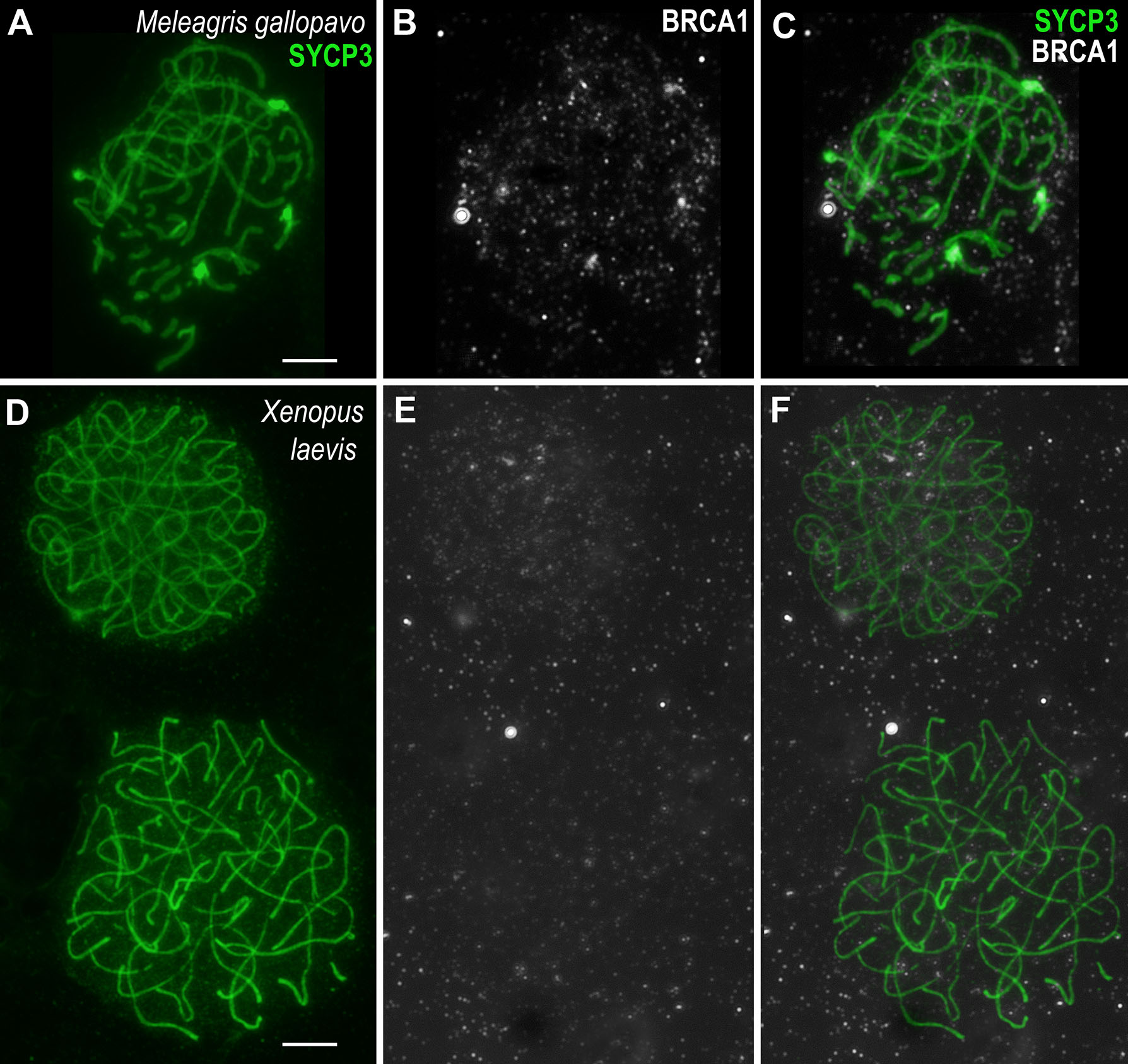


**Figure S8.** Bird and amphibia spermatocytes. SCs were immunostained using antibodies to SYCP3 protein (green), and BRCA1 ubiquitin ligase using antibodies to BRCA1 (white). (A-С) Turkey *Meleagris gallopavo*. Some of the long chromosomes have unpaired/asynaptic regions in the zygotene spermatocyte (top part of nucleus). (D-F) Frog *Xenopus laevis*. Two spermatocytes are at different substages of prophase I. There are no specific BRCA1 signals associated with SCs for both species. Nonspecific BRCA1 signals are visible in the nucleoplasm for both species. Scale bar = 5 µm


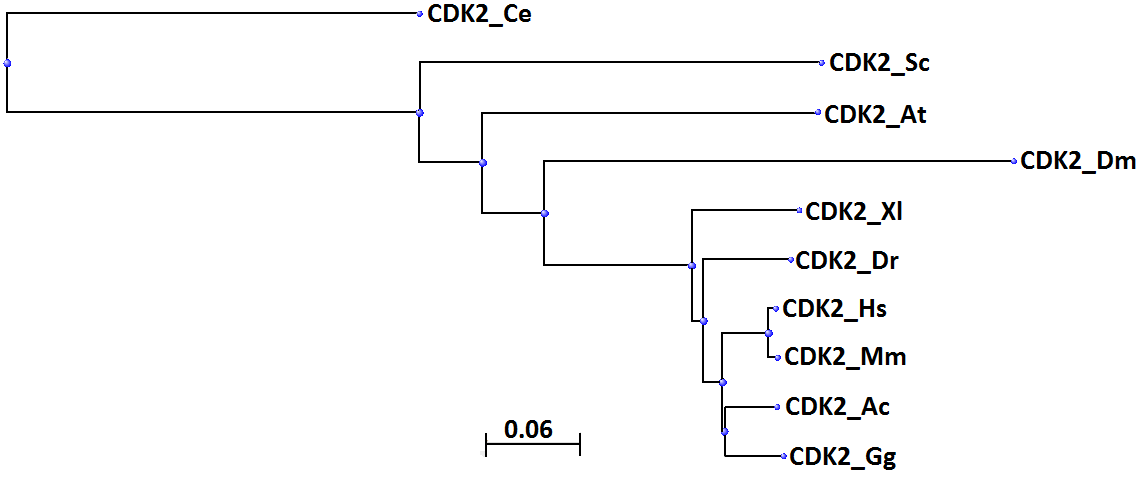


**Figure S9.** Unrooted phylogenetic tree (Neighbor Joining algorithm) for CDK2 orthologs from proteomes representing a wide range of eukaryotes: human (*H. sapiens*, Hs), mouse (*M. musculus*, Mm) (mammals); chicken (*G. gallus*, Gg) (birds); green anole (*А. carolinensis*, Ac) (reptile); clawed frog (*X. laevis*, Xl) (amphibia); zebrafish (*D. rerio*, Dr) (bony fish); fruit fly (*D. melanogaster*, Dm) (insects); roundworm (*C. elegans*, Ce) (nematodes); yeast (*S. cerevisiae*, Sc) (fungi); and Arabidopsis (*A. thaliana*, At) (plants). The evolutionary distance between two sequences was modeled as expected fraction of amino acid substitutions per site given the fraction of mismatched amino acids in the aligned region (according to Grishin, 1995).


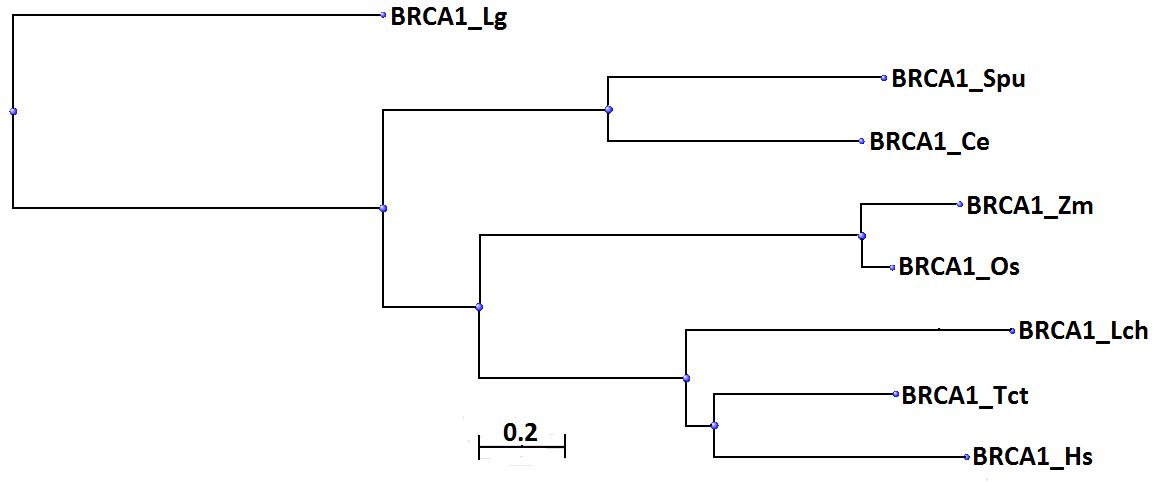


**Figure S10.** Unrooted phylogenetic tree (Neighbor Joining algorithm) for BRCA1 proteins of different eukaryotes: human (Hs) (mammals); turtle (Tct) (reptiles); coelacanth (Lch) (bony fish); owl limpet (Lg) (mollusks); purple sea urchin (Spu) (echinoderms); roundworm (Ce) (nematodes); maize (Zm), rice (Os) (plants). The evolutionary distance between two sequences was modeled as expected fraction of amino acid substitutions per site given the fraction of mismatched amino acids in the aligned region (according to Grishin, 1995).
